# Detection of Gaseous Mercuric Halides Using Acetate and Iodide Chemical Ionization Mass Spectrometry

**DOI:** 10.1021/acs.analchem.5c07120

**Published:** 2026-02-25

**Authors:** Mohammad Borna Bahramsari, Alexei F. Khalizov

**Affiliations:** † Department of Chemistry and Environmental Science, 5965 New Jersey Institute of Technology, Newark, New Jersey 07102, United States; ‡ Department of Chemical and Materials Engineering, New Jersey Institute of Technology, Newark, New Jersey 07102, United States

## Abstract

Predicting the fate of gaseous oxidized mercury (GOM) in the atmosphere remains a challenge because no direct analytical methods exist to determine its molecular speciation. Conventional preconcentration techniques produce speciation losses and induce ligand exchange, obscuring the original composition of GOM. Direct analysis by chemical ionization mass spectrometry (CIMS) offers a path toward addressing this analytical challenge, but its success depends critically on the use of an appropriate ion–molecule chemistry. Previously, we showed the applicability of the negative-ion CIMS for detection of gaseous HgCl_2_. Here, we demonstrate the first systematic evaluation of the detection of gaseous mercuric halides (HgCl_2_, HgBr_2_, and HgI_2_) by acetate (CH_3_COO^–^·CH_3_COOH) and iodide (I^–^) reagent ions, which have been successfully used in the past for the detection of atmospheric trace chemicals. As soft Lewis bases, these reagent ions react promptly with mercuric halides through either complexation or ligand switching. Using a calibrated permeation source, we establish the sensitivity and limit of detection (LOD) for HgBr_2_ with these and previously reported reagent ions. Measured sensitivities range from 3.8 to 21.0 cps/ppbv (for 1 Mcps reagent ion signal), and in the case of ligand switching, they agree with theoretical sensitivities. The current LOD of 53–268 pptv, obtained at a 2.5 Torr ion–molecule reaction pressure, is sufficient for laboratory studies of Hg chemistry and speciating GOM from combustion flue gases. Operating at higher ion–molecule reaction pressures will significantly enhance ionization efficiency, extending the applicability of CIMS toward direct detection of GOM in the atmosphere.

## Graphical abstract

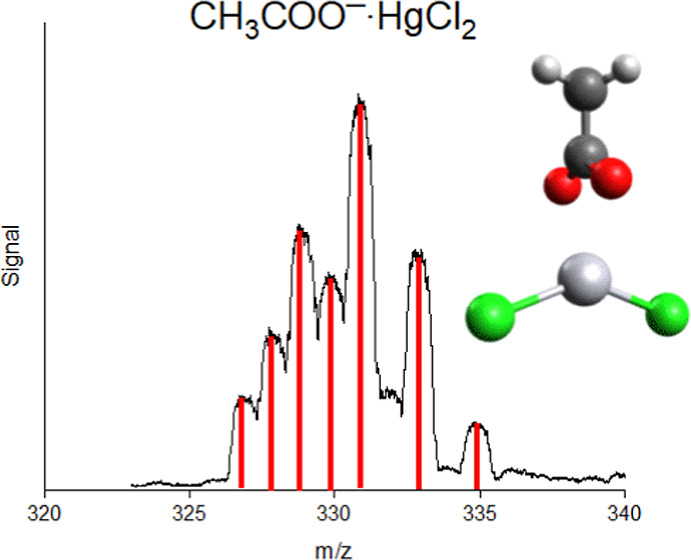

## 1 Introduction

Mercury is a global, persistent neurotoxic pollutant, and its concentration in the atmosphere has increased 7 times due to anthropogenic activities since preindustrial times.^1^ In the atmosphere, it can be present as elemental mercury (gaseous elemental mercury, GEM), oxidized mercury (gaseous oxidized mercury, GOM), and particle-bound mercury (PBM).^2^ In contrast to GEM, which can persist and be transported globally for 0.5–2 years,^3^ GOM and PBM are removed more rapidly due to their higher reactivity and solubility.^4–7^ Evaluating the deposition of these forms is critical, as oxidized mercury in water and soil can be converted into methylmercury,^8,9^ which bioaccumulates and biomagnifies to high levels in fish, an important protein source for humans.^10,11^ The evaluation of the fate of GOM remains challenging because its exact chemical composition is not well-defined. In most cases, the identities of oxidized mercury species are inferred from theoretical considerations rather than confirmed by direct experimental evidence.^12–15^

Current methods for chemical speciation of GOM involve its preconcentration using appropriate media such as cation exchange membranes, nylon membranes, metal oxide glasses, and custom-made collectors.^16,17^ The preconcentrated samples are thermally desorbed to release either the original mercuric compounds or elemental mercury, which are analyzed using various methods. For instance, mass spectrometry-based techniques, such as a commercial atmospheric pressure chemical ionization mass spectrometer (APCI-MS) and a gas chromatograph coupled with an electron impact ionization mass spectrometer (GC–MS), have been used to detect preconcentrated HgCl_2_ and HgBr_2_.^18,19^ In contrast to explicit speciation of the evolving Hg­(II) by MS-based techniques, in the pyrolysis-based methods the chemical speciation is lost, and so they rely on an indirect identification based on the comparison against pure Hg­(II) standards. Specifically, the collected mercuric compounds are desorbed under a controlled temperature ramp, completely pyrolyzed to elemental mercury, and subsequently analyzed using a Tekran analyzer. For chemical speciation, the thermal desorption profiles of field samples are deconvoluted by comparing them with reference profiles obtained from the thermal desorption of permeation-derived pure GOM compounds, including HgBr_2_, HgCl_2_, Hg­(NO_3_)_2_·H_2_O, and HgSO_4_.^20–23^ A major disadvantage of all preconcentration techniques is that subsequent thermal desorption can introduce artifacts, e.g., many proposed oxidized mercury species (e.g., BrHgONO, BrHgONO_2_, BrHgOH, and BrHgOOH) may be labile, have low volatility, or undergo exchange reactions with other chemicals upon collection,^24^ thereby altering the original chemical speciation. Unsurprisingly, only mercuric halides were successfully detected using the combination of preconcentration coupled with thermal desorption and mass spectrometry analysis.^16,17^ Furthermore, preconcentration is inapplicable for the detection of reactive species, such as free radicals, making preconcentration-based methods unsuitable for kinetic and mechanistic studies of transient intermediates.

Chemical ionization mass spectrometry (CIMS) can be used to ionize and analyze neutral molecules directly from the gas phase, offering a way for chemical speciation and quantification of both stable and transient GOM molecules in laboratory and field studies.^25,26^ In CIMS, ionization occurs through an ion–molecule reaction (IMR) of a gaseous analyte with a reagent ion produced in corona discharge or using a radioactive or X-ray source. The sensitivity and limit of detection of CIMS depend on the IMR mechanism, reaction pressure, cleanliness of the system, and other factors, giving rise to various method implementations that have been used for the direct detection of various atmospheric constituents.^27–29^ Our research group has previously explored the use of three reagent ions, SF_6_^–^, NO_3_^–^·HNO_3_, and CO_*n*_^–^ (*n* = 3 and 4), reporting their relative detection sensitivities toward HgCl_2_.^30^ Later, we employed two of those ions, SF_6_^–^ and NO_3_^–^·HNO_3_, for the investigations of the heterogeneous uptake^31,32^ and surface-catalyzed exchange reactions^30^ of mercuric halides. While SF_6_^–^ showed a significantly higher detection sensitivity than NO_3_^–^·HNO_3_, its performance was affected by water vapor, prompting the use of the nitrate complex ion in the experiments where water vapor concentration was elevated.^31^ Other studies also reported the interfering effect of water on SF_6_^–^, caused by the irreversible reactions to form other ion products and limiting the use of SF_6_^–^ in atmospheric measurements.^31,32^ Unlike SF_6_^–^, ions such as iodide (I^–^) and acetate (CH_3_COO^–^) undergo only a reversible reaction with water, making them suitable for atmospheric detection of trace organic and inorganic compounds, including hyperoxides, organic and inorganic acids, and vaporized particle constituents in secondary organic aerosols.^33–37^

This work evaluates the suitability of the iodide and acetate reagent ions for the detection of gaseous mercuric halides HgX_2_ (where X is Cl, Br, or I) in the laboratory and potentially for atmospheric measurements. We investigate the IMR mechanism, and using a calibrated HgBr_2_ source, we establish the absolute sensitivity and the limit of detection for these reagent ions, as well as for SF_6_^–^ used previously.

## 2 Methods

### 2.1 Chemicals

Mercuric chloride (HgCl_2_, Honeywell, >99.5%), mercuric bromide (HgBr_2_, Alfa Aesar, >99%), and mercuric iodide (HgI_2_, Sigma-Aldrich, >99%) were used as surrogates for GOM. Iodomethane (CH_3_I, Alfa Aesar, >99.5%), trifluoro iodomethane (CF_3_I, Sigma-Aldrich, 99%), acetic anhydride (C_4_H_6_O_3_, Sigma-Aldrich, >99%), and SF_6_ (15% in argon, Airgas) were used as precursors for the generation of reagent ions. Helium and nitrogen compressed gases were of ultrahigh purity (UHP) and provided by Airgas.

### 2.2 Ion Drift-Chemical Ionization Mass Spectrometer

The ion drift-chemical ionization mass spectrometer (ID-CIMS) used in this work is similar to that described in our previous studies.^24,30,38,39^ It consists of an ion drift tube (DT) and a collision deactivation chamber (CDC) connected to a differentially pumped vacuum chamber that houses a quadrupole mass spectrometer (Figure S1). Chemical ionization takes place in the ion drift tube upon mixing of a gas flow containing neutral analyte with a flow containing reagent ion produced from an appropriate precursor in the afterglow of a negative corona discharge. The drift tube is a stack of five stainless steel rings (1/2 in. aperture and 1/8 in. thickness) separated by five static-dissipating fluoropolymer insulators (1 in. aperture and 3/4 in. thickness) sealed by Viton O-rings. The CDC is a stack of four stainless steel ring electrodes (1/2 in. aperture and 1/16 in. thickness) and four static dissipating fluoropolymer insulators (1/2 in. aperture and 3/8 in. thickness). In this work, the pinhole between DT and CDC is removed to maintain the same 1–3 Torr pressure in both parts, essentially resulting in a longer DT. The ring electrodes in DT and CDC are electrically connected via resistors, and a small electric current is sent through the resistors to develop a predefined voltage drop along the electrode stack. The DT resistors are fixed at 1 MΩ. The CDC resistors are single-turn potentiometers adjusted to about 30% of their maximum value of 2 MΩ to set a uniform electric field through both regions, DT and CDC. The electric field focuses the ions in a tight beam and also allows control of the ion–molecule reaction time and ion clustering.^40^ At the end of CDC, ions are focused through a 0.5 mm pinhole into the differentially pumped chamber, where most of the neutral gas molecules are removed in the first compartment while the ions are refocused and guided by lenses into the second compartment, where they are detected using a quadrupole mass filter with a counting channeltron electron multiplier. Compared to our previous work,^30^ the corona discharge–DT region, pumping, and quadrupole system were upgraded to improve performance, as described below.

First, we changed the location where the reagent ions are mixed with the analyte. Previously, the mixing point was inside DT, where the analyte flow entered DT coaxially through the front ring electrode and the reagent ion flow entered at a right angle to DT through the fluoropolymer insulator between the first and second ring electrodes. Such a configuration required the use of a strong electric field to refocus the ions in a tight beam to maintain a high ion transmission. In the new configuration (Figure S1), while the corona discharge source is kept transverse to the drift tube axis, the reagent ion and analyte flows are mixed in a glass tee and only then introduced axially into DT. By moving the mixing region outside of DT, this new design allowed achieving sufficient ion beam focusing even with electric fields as low as 5 V cm^–1^, resulting in longer ion–molecule reaction times. It should be noted that positioning the corona discharge source coaxially with DT results in a significant mass-independent background signal (100–150 kcps) when detecting negative ions despite the use of an off-axis electron multiplier. The shortwave ultraviolet emitted by corona discharge enters the vacuum chamber through the pinhole and strikes the metal parts, causing the photoelectric effect. The emitted electrons are then attracted to the electron multiplier entrance, which is held at a high positive potential (+3 kV) during negative ion detection, producing secondary electrons that result in a high background count. Additionally, we found that placing the corona discharge source at a longer distance from the DT axis significantly reduces the mass-independent background count without affecting the reagent ion signal intensity. The mass-independent background is caused by the shortwave ultraviolet emitted by the metastables emerging from the corona discharge and reaching DT.

Second, we found that optimum refocusing of ions in DT can be achieved by keeping the first and second ring electrodes at the same potential, resulting in zero electric field in that region. As shown by Hanson et al.^40^ using SIMION simulations, the fringe electric field penetrating into this zero-field region from the region between the second and third ring electrodes helps to refocus the ions into a tight beam.

Third, two turbopumps were added to the first chamber to triple the pumping speed from 250 L/s to 750 L/s. Those pumps are an Agilent TwisTorr 304 FS and a split-flow Agilent TwisTorr 305 SF. The two regular turbo pumps are backed by a single Agilent DS302 rotary vane pump. The split flow turbo pump is backed by an Agilent DS402 rotary vane pump. In future applications, the split flow turbopump will be used to provide 10 L/s pumping of the CDC region, but currently the side port is sealed off. A larger pumping speed allows increasing the maximum DT pressure from 3 to 5 Torr concurrently with enlarging the sampling pinhole diameter from 0.3 mm to 0.5 mm to allow more ions to enter the detection chamber. The rear chamber, holding the quadrupole and multiplier, is pumped by a single Agilent TwisTorr 304 FS backed by a single Agilent DS302 rotary vane pump, allowing it to maintain a 10^–6^ Torr vacuum with a full gas load.

Fourth, the original old-generation 19 mm quadrupole was replaced with a 19 mm segmented quadrupole equipped with prefilter and postfilter rods. These RF-only rods reduce the fringing electric field at the entrance and exit of the mass filter, improving both ion transmission and resolving power.

### 2.3 Sources of Gaseous Mercuric Halides

Three different sources were used to generate gaseous mercuric halides: a commercial permeation tube, a glass tube packed with small glass beads coated by solid mercuric halides, and a glass tube with a thin coat of solid mercuric halides on its inner wall. A detailed description of each source is provided below.

The permeation source was based on a certified, gravimetrically calibrated permeation tube supplied by VICI Metronics, producing 556.3 ng min^–1^ HgBr_2_ at 100 °C and atmospheric pressure. The 9.5 mm diameter and 8 cm length permeation tube was placed at the bottom of one arm of a temperature-controlled U-shaped glass tube of 1.7 cm diameter and 20 cm height. The other arm was filled with 0.6 cm diameter spherical glass beads to ensure a fast temperature equilibration of the gas flow. The two arms of the U-tube are divided at the bottom by a glass separator with multiple holes. The HgBr_2_-laden gas flow leaving the U-tube was diluted with a flow of nitrogen, using a tee adaptor (Figure S2). A critical orifice installed between the second tee and ID-CIMS ensured a constant sampling flow of 300 sccm. To maintain a uniform temperature, the U-tube was encased in a thin, pliable copper sheet, and the space between the glass and copper was filled with copper wool to ensure efficient heat transport. The entire assembly was wrapped with a heating tape and two layers of fiberglass insulation. A constant temperature was maintained using a PID controller with a thermocouple attached to the outside glass wall next to the region where the permeation tube was located. Separate experiments showed that the temperature in the arm holding the permeation tube was within 0.5 °C of the target temperature (Figure S3). The mixing ratio of HgBr_2_ was varied between 0.31 and 4.2 ppbv by setting the carrier flow through the U-tube to 500 sccm and adjusting the dilution flow between 4 and 60 slpm. The lower limit on the carrier flow is imposed at about 360 sccm to prevent condensation of HgBr_2_ upon cooling of the gas to room temperature.

Although the permeation tube produces a known mercury output, it is not a convenient source for everyday experimental work, as it must be continuously purged by a flow of gas at ambient pressure and at a precisely controlled elevated temperature (100 °C). Hence, most qualitative measurements were performed using the other two sources, whereas the permeation source was used only to determine the sensitivity of the ID-CIMS toward HgBr_2_ with different reagent ions. The first alternative source consisted of glass beads (0.5 mm diameter, Cat. No. 11079105, BioSpec Products) coated with a solid mercuric halide and packed inside a 1/4″ outer diameter (OD) glass tube between a cotton wool plug upstream and a porous polyethylene plug (made from a 1/8″ OD rod, 15–40 μm pore size, Porex-5520, Interstate Specialty Products) downstream (Figure S4). The second alternative source was made of a 1/4″ OD glass tube coated on the inside with a solid mercuric halide layer. A filter holder (Savillex, 25 mm Single Stage Filter Assembly) loaded with a PTFE membrane (Savillex, 25 mm Filter Membrane, 5–6 μm pore size) was connected downstream to prevent accidentally detached mercuric halide particles from reaching the drift tube (Figure S4). The first source performed without any reduction in its output over a year but created a significant pressure drop, up to 20 Torr at a 4 sscm flow. The second source produced only a small pressure drop (1.2 Torr) with the same flow but had to be recharged after a few days due to the smaller amount of mercuric halide that could be securely deposited on the tubing wall. Stability of both sources was verified by repeated measurements of the HgX_2_ signal under identical operating conditions, with no systematic drift observed within experimental uncertainty.

### 2.4 Calculations

Density Functional Theory (DFT) calculations were performed using Gaussian 16 (Revision C.01).^41^ Geometry optimizations and vibrational frequency calculations were carried out at the M06-2X^42^ level of theory using the aug-cc-pVTZ^43,44^ basis set for H, C, O, and Cl and the aug-cc-pVTZ-PP^45^ basis set for the outer electrons of Hg, Br, and I. M06-2X is empirically parameterized to account for medium-range electron correlation, which is particularly relevant for ion–molecule interactions, and has been previously used by our research group and others for mercury-containing molecules, showing reliable performance.^13,14,30,46–48^ To account for scalar relativistic effects in Br, I, and Hg, the Stuttgart/Köln effective core potentials (ECPs) were used, replacing the 10, 28, and 60 innermost electrons, respectively.^49–51^ Although CCSD­(T) single-point energy calculations improve accuracy by 1–5 kcal/mol,^30,52^ the larger errors occur mostly for nonisogyric reactions where the multiplicity is not preserved, such as when calculating electron affinity. Most reactions of interest involve clustering between a closed-shell neutral molecule and a radical anion. In electron affinity calculations, several species exhibit open-shell electronic configurations. Closed-shell species were treated using restricted DFT, whereas open-shell radical anions and the resulting ion–molecule complexes were treated using unrestricted DFT. For all open-shell systems, wave function stability checks were performed after geometry optimizations and frequency calculations. Geometry optimizations were performed without imposing symmetry constraints. Binding energies of the ion–molecule complexes were estimated from total electronic energies obtained at the DFT level.

The Ion Mobility Spectrometry Suite v1.13 (IMoS) software was used to calculate the reduced ion mobility of reagent ions under standard ambient temperature and pressure (SATP) conditions.^53,54^ The input file for IMoS included the Cartesian coordinates, van der Waals radii, and Mulliken charges of atoms in molecules obtained from DFT calculations (Table S2). The trajectory method with Lennard-Jones and quadrupole potentials was applied using N_2_ gas. Default Lennard-Jones parameters and N_2_ quadrupole parameters implemented in IMoS were used without modification (Table S3). To validate the accuracy of this method, the reduced ion mobilities of SF_6_^–^, CO_3_^–^, NO_3_^–^·HNO_3_, and H_3_O^+^ were calculated and compared with experimental results. For all ions, the relative error was less than 10% (Table 1).

**Table 1:** Reduced Ion Mobilities (cm^2^ V^–1^ s^–1^) Used for Calculation of Ion Velocities and Ion–Molecule Reaction Times

ion	calculated^57^	literature	relative error %
SF_6_^–^	2.17	1.99 ^58^	9.0
CO_3_^–^	2.39	2.55 ^59^	6.2
NO_3_^–^·HNO_3_	2.00	1.86 ^60^	7.5
H_3_O^+^	2.95	2.76 ^61^	6.8
CH_3_COO^–^·CH_3_COOH	1.72	n/a	
I^–^	2.13	n/a	

Ion–molecule reaction rates were calculated using the Average Dipole Orientation (ADO) theory, which is based on the collision rate between reagent ions and analyte molecules.^55,56^ The ADO rate constant is given by

1 $$k_{ADO}=\left(\frac{2\pi q}{\sqrt{\mu }}\right)\left[\sqrt{\alpha }+C\mu_{D}\sqrt{\left(\frac{2}{\pi k_{B}T}\right)}\right]$$

where μ is the reduced mass of the analyte and reagent ion, *q* is the charge of the reagent ion, *k*_B_ is the Boltzmann constant, α is the isentropic component of the polarizability tensor, and μ_D_ is the dipole moment; μ_D_ and α were obtained by DFT calculation of the analyte molecules. *C* is a parameter, ranging between 0 and 1, which describes the effectiveness of the charge locking in the dipole. Since the dipole moment of mercuric halides is zero, eq 1 reduces to the Langevin equation

2 $$k_{L}=\left(\frac{2\pi q}{\sqrt{\mu }}\right)\sqrt{\alpha }$$

## 3 Results and Discussion

### 3.1 Ion Chemistry

To study ion–molecule reactions, we selected three mercuric halides, HgCl_2_, HgBr_2_, and HgI_2_, as surrogates for GOM because of their commercial availability. The relatively high saturation vapor pressures of these chemicals at room temperature allowed the construction of simple sources with stable outputs in the range of 396–5911 ng/h, as estimated from the measured sensitivities (Section 3.2). Although these halides are nonpolar, unlike proposed atmospheric GOM molecules such as BrHgNO_2_,^13^ varying the halogen substituent X in HgX_2_ enables exploration of the effects of polarizability and Hg–X bond strength on the efficiency of ion–molecule reactions. From HgCl_2_ to HgI_2_, polarizability increases while Hg–X bond strength decreases. We used three methods of delivery of gaseous mercuric halides: two semiquantitative methods rely on the saturation vapor pressure of HgX_2_, and a fully quantitative method is based on a calibrated HgBr_2_ permeation source.

Ion–molecule reactions of HgX_2_ with the iodide and acetate reagent ion families can proceed through three pathways: electron transfer, ion clustering, and ion transfer. According to DFT calculations (Table 2), the energy required to remove an electron (−Δ*H*) from all reagent ions is greater than the electron affinities of all mercuric halides, where the calculated electron affinities agree with values available in the literature. Therefore, electron transfer is not a thermodynamically feasible pathway. However, ion clustering and ion transfer reactions between reagent ions and mercuric halides are all exothermic (Tables 3 and 4). Notably, the enthalpy changes between different mercuric halides are close to each other for the same reagent ion, within 1 kcal/mol. Differences in Gibbs free energy are somewhat larger (within 3 kcal/mol) and arise due to variations in the reaction entropy between different mercuric halides (Tables 3 and 4). DFT calculations also indicate that acetate forms a significantly stronger complex with HgX_2_ compared to iodide. The bond dissociation enthalpies for acetate clusters with mercuric halides are in the range of 42–43 kcal/mol, whereas for iodide, they are in the range of 35–36 kcal/mol. Optimized structures of iodide and acetate clusters with mercuric halides are shown in Figure 1. Upon complex formation, significant geometric transformations take place in HgX_2_: the initial X–Hg–X 180° angle decreases to 117–140°, depending on the ion, and the Hg–X bond distances elongate by 0.13–0.18 Å.

**Table 2:** Calculated and Literature Values of Electron Affinities (EA) of Reagent Ions and Mercuric Halides

		Δ*H* (kcal/mol)	
#	reaction	calculation	literature
1.1	$I+e^{-}\to I^{-}$	–70.62	–70.54 ^62^
1.2	$I_{2}+e^{-}\to I_{2}^{-}$	–61.91	–55.34 ^63^
1.3	$I_{3}+e^{-}\to I_{3}^{-}$	–98.26	–97.45 ^64^
1.4	${\left(CH_{3}CO\right)}_{2}O+e^{-}\to {{\left(CH_{3}CO\right)}_{2}O}^{-}$	11.51	n/a
1.5	$CH_{3}COO+e^{-}\to {CH_{3}COO}^{-}$	–78.40	–74.94 ^65^
1.6	$Hg{Cl}_{2}+e^{-}\to {Hg{Cl}_{2}}^{-}$	–42.12	–36.67 ^66^
1.7	$Hg{Br}_{2}+e^{-}\to {Hg{Br}_{2}}^{-}$	–44.45	–37.36 ^66^
1.8	$HgI_{2}+e^{-}\to {HgI_{2}}^{-}$	–46.41	n/a

**Table 3:** Enthalpies and Free Energies of Reactions between Mercuric Halides and the Acetate Family of Reagent Ions Produced Using (CH_3_CO)_2_O as a Precursor

#	reaction	Δ*H* (kcal/mol)	Δ*G* (kcal/mol)
2.1	$Hg{Cl}_{2}+{CH_{3}COO}^{-}\to {Hg{Cl}_{2}\cdot CH_{3}COO}^{-}$	–42.73	–37.52
2.2	$Hg{Br}_{2}+{CH_{3}COO}^{-}\to {Hg{Br}_{2}\cdot CH_{3}COO}^{-}$	–43.35	–35.46
2.3	$HgI_{2}+{CH_{3}COO}^{-}\to {HgI_{2}\cdot CH_{3}COO}^{-}$	–43.77	–38.73
2.4	$Hg{Cl}_{2}+{CH_{3}COO}^{-}\cdot CH_{3}COOH\to {Hg{Cl}_{2}\cdot CH_{3}COO}^{-}+CH_{3}COOH$	–10.43	–14.72
2.5	$Hg{Br}_{2}+{CH_{3}COO}^{-}\cdot CH_{3}COOH\to {Hg{Br}_{2}\cdot CH_{3}COO}^{-}+CH_{3}COOH$	–11.04	–12.67
2.6	$HgI_{2}+{CH_{3}COO}^{-}\cdot CH_{3}COOH\to {HgI_{2}\cdot CH_{3}COO}^{-}+CH_{3}COOH$	–11.46	–15.94

**Table 4:** Enthalpies and Free Energies of Reactions between Mercuric Halides and the Iodide Family of Reagent Ions Produced Using CF_3_I as a Precursor

#	reaction	Δ*H* (kcal/mol)	Δ*G* (kcal/mol)
3.1	HgCl_2_ + I^–^ → HgCl_2_I^–^	–35.79	–34.28
3.2	HgBr_2_ + I^–^ → HgBr_2_I^–^	–36.36	–34.21
3.3	HgI_2_ + I^–^ → HgI_2_I^–^	–36.48	–34.07
3.4a	HgCl_2_ + I_2_^–^ → HgCl_2_I_2_^–^	–23.15	–19.38
3.4b	HgCl_2_ + I_2_^–^ → HgCl_2_I^–^ + I	–10.70	–14.12
3.5a	HgBr_2_ + I_2_^–^ → HgBr_2_I_2_^–^	–23.43	–17.34
3.5b	HgBr_2_ + I_2_^–^ → HgBr_2_I^–^ + I	–11.27	–11.81
3.6a	HgI_2_ + I_2_^–^ → HgI_2_I_2_^–^	–23.41	–20.07
3.6b	HgI_2_ + I_2_^–^ → HgI_2_I^–^ + I	–11.38	–13.91
3.7a	HgCl_2_ + I_3_^–^ → HgCl_2_I_3_^–^	–16.25	–16.47
3.7b	HgCl_2_ + I_3_^–^ → HgCl_2_I^–^ + I_2_	–4.65	–13.28
3.8a	HgBr_2_ + I_3_^–^ → HgBr_2_I_3_^–^	–16.39	–15.62
3.8b	HgBr_2_ + I_3_^–^ → HgBr_2_I^–^ + I_2_	–5.22	–10.97
3.9a	HgI_2_ + I_3_^–^ → HgI_2_I_3_^–^	–16.39	–16.81
3.9b	HgI_2_ + I_3_^–^ → HgI_2_I^–^ + I_2_	–5.34	–13.07
3.13	HgCl_2_ + CF_3_I_2_^–^ → HgCl_2_I^–^ + CF_3_I	–17.85	–22.85
3.14	HgBr_2_ + CF_3_I_2_^–^ → HgBr_2_I^–^ + CF_3_I	–18.42	–20.54
3.15	HgI_2_ + CF_3_I_2_^–^ → HgI_2_I^–^ + CF_3_I	–18.54	–22.64

**Figure 1:**
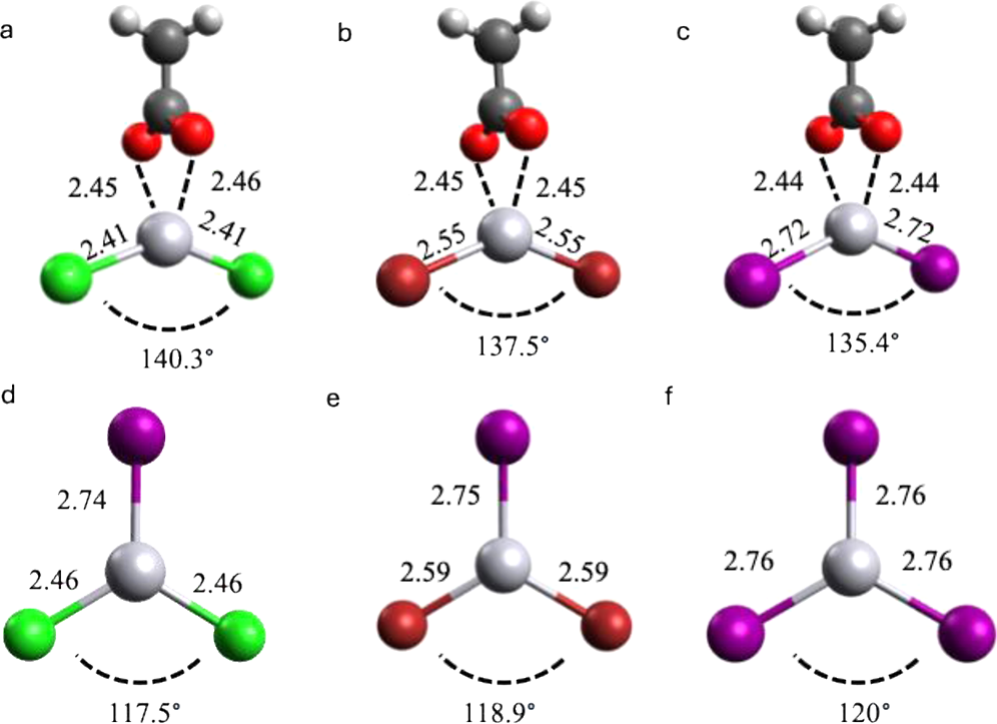
Calculated structures of the experimentally observed products of reactions of the three neutrals (HgCl_2_, HgBr_2_, and HgI_2_) with two reagent ions, CH_3_COO^–^·CH_3_COOH and I^–^. Panels: (a) HgCl_2_·CH_3_COO^–^ (C_1_/C_s_), (b) HgBr_2_·CH_3_COO^–^ (C_1_/C_s_), (c) HgI_2_·CH_3_COO^–^ (C_1_/C_s_), (d) HgCl_2_I^–^ (C_1_/C_2v_), (e) HgBr_2_I^–^ (C_1_/C_2v_), and (f) HgI_2_I^–^ (C_3v_/C_3v_). Atom colors: Hg (gray), C (dark gray), O (red), H (white), Cl (green), Br (brownish red), and I (violet). All reported internuclear distances are given in angstroms (Å). Geometry optimizations were performed without symmetry constraints. The symmetries are given in parentheses after the complexes, where the first symbol denotes the point group reported by Gaussian, whereas the second symbol denotes an approximate symmetry inferred from the optimized geometries.

Depending on the drift tube’s electric field and the concentration of reagent ion precursor, the following reagent ions were identified in our experimental system: CH_3_I produced I^–^, I_2_^–^, and I_3_^–^; CF_3_I produced I^–^, I_2_^–^, CF_3_I_2_^–^, and I_3_^–^; and (CH_3_CO)_2_O produced CH_3_COO^–^·CH_3_COOH, CH_3_COO^–^, and (CH_3_CO)_2_O^–^. The electric field strength was optimized to the lowest possible value, allowing the focusing of ions into a narrow beam, thereby maximizing product-ion signal output while maintaining high reagent-ion signal intensity. Figures S5 and S6 show the detuned spectrum of I^–^ (*m*/*z* = 127) in the CF_3_I/N_2_ and CH_3_I/N_2_ systems, respectively. Detuning was required to prevent the saturation of the multiplier and was achieved by adjusting the voltage of the quadrupole exit lens; this method allowed the broadest control of the ion intensity and the lowest mass discrimination. However, detuning affected the ion energy in the quadrupole, resulting in the peak shape deviating from a Gaussian profile and appearing distorted. The signal intensity of iodide at a full multiplier voltage (2100 V) was estimated indirectly, using the experiments described in Section 3.2. In addition to the bare iodide, other ions were formed from CF_3_I and CH_3_I in relatively high concentrations. Figure S7 shows the mass spectra of the ions produced from CF_3_I, including I_2_^–^ (*m*/*z* = 254 amu), I_3_^–^ (*m*/*z* = 381 amu), and CF_3_I_2_^–^ (*m*/*z* = 323 amu) with signal intensities of 150, 350, and 750 kcps, respectively. The relatively strong signal of CF_3_I_2_^–^ is likely due to the high concentration of CF_3_I in the system, resulting in its clustering with I^–^. Figure S8 shows the mass spectra of the ions produced from CH_3_I, including I_2_^–^ (*m*/*z* = 254 amu), I_3_^–^ (*m*/*z* = 381 amu), and an unknown ion at *m*/*z* = 280 amu with signal intensities of 40, 60, and 100 kcps, respectively. Figure S9 presents the mass spectra of the ions observed using (CH_3_CO)_2_O as a precursor. The signal intensities of CH_3_COO^–^ (*m*/*z* = 59 amu), (CH_3_CO)_2_O^–^ (*m*/*z* = 102 amu), and CH_3_COO^–^·CH_3_COOH (*m*/*z* = 199 amu) were 200, 250, and 1100 kcps, respectively. When possible, in most of our experiments, the parameters of the system were adjusted to have I^–^ and CH_3_COO^–^·CH_3_COOH as the major reagent ions.

When mercuric halides were introduced into the drift tube, the only observed ion products in the iodide and acetate systems were HgX_2_·I^–^ and HgX_2_·CH_3_COO^–^, respectively (Figure 2). The signal intensities of the iodide complexes HgCl_2_·I^–^ (*m*/*z* = 395–405 amu), HgBr_2_·I^–^ (*m*/*z* = 483–493 amu), and HgI_2_·I^–^ (*m*/*z* = 577–585 amu) were 14.0, 1.1, and 0.6 kcps, respectively. The signal intensities of the acetate complexes HgCl_2_·CH_3_COO^–^ (*m*/*z* = 325–337 amu), HgBr_2_·CH_3_COO^–^ (*m*/*z* = 413–426 amu), and HgI_2_·CH_3_COO^–^ (*m*/*z* = 509–519 amu) were 25.0, 1.4, and 1.4 kcps, respectively. The difference in signal intensities between various mercuric halides for the same reagent ion can be attributed to differences in their saturation vapor pressures, producing different concentrations of these halides in the drift tube. The difference in signal intensities between the iodide and acetate systems for the same mercuric halides is mostly due to the different product ion stabilities, as described in Section 3.2.

**Figure 2:**
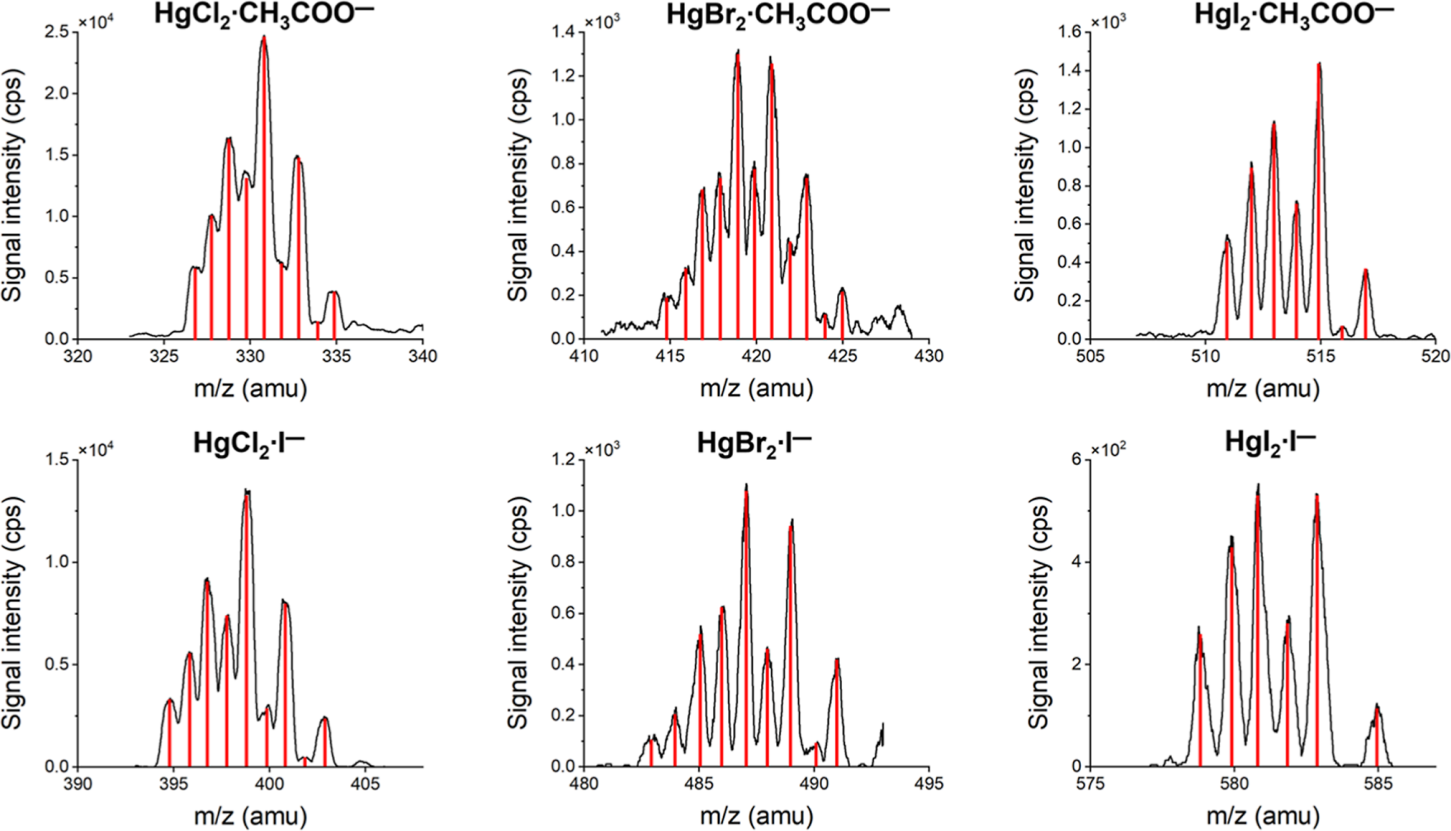
Mass spectra of product ions produced in the reaction of HgCl_2_, HgBr_2_, and HgI_2_ neutrals with CH_3_COO^–^·CH_3_COOH and I^–^ reagent ions. The black curves are for the experimental spectra, and the red sticks indicate the theoretical spectra.

When comparing the product ions observed in the experiments against the predictions based on the DFT calculations, one can see the absence of complexes such as HgX_2_·I_2_^–^ and HgX_2_·I_3_^–^. We surmise that these complexes are unstable under the conditions of our experiment. Either they are not thermolyzed and dissociate immediately after formation, or they dissociate during collisions promoted by the electric field in the drift tube or near the pinhole separating the drift tube from the lower pressure region in CIMS. As described in Section 3.2, even HgX_2_·I^–^ complexes, which are bonded by about 36 kcal/mol, were not fully thermalized, partially dissociating at the 2–3 Torr pressure of the drift tube. By taking the difference between *a* and *b* channels in Table 4, one can see that the elimination of the I and I_2_ neutrals from HgX_2_·I_2_^–^ and HgX_2_·I_3_^–^ requires only 11–12 kcal/mol. Thus, the decomposition of these complexes due to inefficient thermalization is plausible. Similarly, no complexes of HgX_2_ with CH_3_COO^–^·CH_3_COOH and (CH_3_CO)_2_O^–^ were observed.

### 3.2 Sensitivity and Limit of Detection

In our previous study,^30^ the sensitivity for detection of HgCl_2_ by several reagent ions has been estimated, assuming that the helium flow passing through a bed made of powdered HgCl_2_ was fully saturated with HgCl_2_ vapor. The resulting experimental sensitivity was found to be significantly lower than theoretical sensitivity, indicating that the saturation assumption may have been invalid. Here, we relied on a gravimetrically calibrated permeation source to produce a gas flow with a known concentration of HgBr_2_. This source was used to measure the sensitivity in the iodide and acetate systems. Additionally, we remeasured the sensitivity with the SF_6_^–^, using HgBr_2_ as the analyte. All calibrations were performed in a 0.3–3.2 ppbv range of HgBr_2_ concentrations, using selected-ion monitoring (SIM). The experimentally measured sensitivities ranged from 17 to 83 cps/ppbv, depending on the reagent ion (Table 5), which was a result of not only different reagent ion signal intensities but also of different product ion stabilities. To make the comparison possible, the sensitivities were normalized to the reagent ion count rate of 1 Mcps, as also shown in Table 5.

**Table 5:** Measured Experimental Sensitivities, Limits of Detection, and Normalized Theoretical Sensitivities for the Detection of HgBr_2_ Using Different Reagent Ions

	reagent ion signal intensity	*S*_exp_	LOD^a^	*S*_exp_^0^	
reagent ion	Mcps	cps ppb^–1^	ppt	cps ppb^–1^ @ 1 Mcps reagent ion	*S*_t_ ^b^/*S*_exp_^0^
CH_3_COO^–^·CH_3_COOH	1.1	22	216	21.0 ± 0.9	1.3
I^–^ ^c^	4.5	17	53	3.8 ± 0.1	6.6
I^–^ ^d^	7.6	18	268	2.3 ± 0.2	10.7
SF_6_^–^	4.5	83	102	18.5 ± 0.5	1.3

a Calculated for a 60 s integration time and assuming a signal-to-noise ratio S/N = 3.

b Theoretical sensitivity normalized to 1 Mcps reagent ion signal intensity.

c Produced from CF_3_I.

d Produced from CH_3_I.

To measure the signal intensity of CH_3_COO^–^·CH_3_COOH during calibration experiments while preventing the saturation of the electron multiplier, the less abundant isotopic peak with an *m*/*z* of 120 was used. Since iodine has only a single isotope, this approach could not be used for I^–^ and instead its signal intensity was calculated from the ratio of detuned to tuned H_2_C_2_O_4_·I^–^ signals, using a small addition of H_2_C_2_O_4_. Since the detuned-to-tuned signal ratio depends on the ion’s mass-to-charge ratio (*m*/*z*), a fit was constructed based on the measurements across a broad spectral range (Figure S10). From the fit, the mass correction coefficient (*C*_mass_) for H_2_C_2_O_4_·I^–^ (*m*/*z* = 217) relative to I^–^ (*m*/*z* = 127) was determined to be 2.02. The tuned iodide signal intensity was then obtained using the following equation,

3 $$I_{T}\left(I^{-}\right)=I_{DT}\left(I^{-}\right)\times C_{mass}\times \frac{I_{T}\left({H_{2}C_{2}O_{4}\cdot I}^{-}\right)}{I_{DT}\left({H_{2}C_{2}O_{4}\cdot I}^{-}\right)}$$

where *I*_T_(I^–^) is the tuned signal intensity of iodide, *I*_DT_(I^–^) is the detuned signal intensity of iodide, *C*_mass_ is the mass correction coefficient H_2_C_2_O_4_·I^–^ (*m*/*z* = 217) relative to I^–^ (*m*/*z* = 127), *I*_T_(H_2_C_2_O_4_·I^–^) is the tuned signal intensity of the oxalic acid cluster with iodide, and *I*_DT_(H_2_C_2_O_4_·I^–^) is the detuned signal intensity of the oxalic acid cluster with iodide.

Oxalic acid was intentionally added to the system to provide a reference for the iodide signal. It was selected because its concentration could be readily controlled and the mass of its product ion (*m*/*z* = 217) was relatively close to that of iodide (*m*/*z* = 127), producing a lower error from mass discrimination. The detuned-to-tuned ratios of H_2_C_2_O_4_·I^–^ were 20.2 and 17.5 for the CF_3_I/N_2_ and CH_3_I/N_2_ systems, respectively, and this ratio was assumed to remain constant under tuned ion transmission conditions. Detuning was performed by changing the quadrupole exit lens from +40 V to −30 V in Merlin Automation software. The detuning method was validated using SF_6_^–^ as the reagent ion, since its signal intensity could be independently calculated from the less abundant (4.4%) isotopic peak at *m*/*z* = 148 amu. When oxalic acid was used as the reference to calculate the signal intensity of the most abundant isotopic peak of SF_6_^–^ (*m*/*z* = 146 amu) based on its isotopic peak at *m*/*z* = 148 amu, the relative error of the tuning method was 3.1%.

Figure 3 shows calibration plots where product ion intensities are normalized per million total reagent ion cps. The plots indicate a good linearity; the observed scatter in data points can be attributed to variations in the mercuric bromide concentration and reagent ion signal. For mercuric bromide, the variability is caused jointly by a ±1 °C temperature oscillation of the PID-controlled heating system that creates a 7–10% variation in the emission rate from the permeation source, flow and pressure variability introduced by mass flow controllers (about 2%), and incomplete signal stabilization caused by wall adsorption/desorption of mercuric bromide when its concentration is increased or decreased. As for the reagent ion, due to the high intensity, its signal was measured indirectly at the end of each concentration point, introducing some uncertainty, as described above. The sensitivities derived from the slopes of the plots are listed in Table 5, decreasing in the order CH_3_COO^–^·CH_3_COOH, SF_6_^–^, I^–^ (generated from CF_3_I), and I^–^ (generated from CH_3_I). The difference between the sensitivities obtained using the iodide produced by the two different precursors arises from the presence of a high concentration of CF_3_I_2_^–^ in the CF_3_I/N_2_ system (Figure S7). Although the signal intensity of CF_3_I_2_^–^ is only about one-sixth that of iodide, it can outcompete the iodide by forming a fully thermalized HgBr_2_·I^–^ in an ion exchange reaction 3.14 (Table 4), where collisional thermalization of the product is not required, as the energy produced upon complex formation is removed in the form of kinetic energy of the two products. On the contrary, in clustering reaction 3.2 between iodide and mercuric bromide, the resulting HgBr_2_·I^–^ complex is roto-vibrationally hot and must be thermalized through collisions with the surrounding gas. Since the pressure in our system was relatively low, the dissociation rate was higher than the thermalization rate (see Section S1 in Supporting Information describing the calculation of the thermalization and decomposition rates), meaning the net collision rate with the bath gas determined the outcome. Consequently, fewer iodide–mercury clusters were detected with CH_3_I, leading to a lower sensitivity where only bare iodide was present (Table 5). Similar results were reported by Thornton et al.,^34^ where the sensitivity of detection of formic acid by I^–^ increased with relative humidity because when I­(H_2_O)^−^ reacted with formic acid, the leaving water molecule carried away a large fraction of the reaction energy, requiring no collisional thermalization of the formic acid–iodide complex.

**Figure 3:**
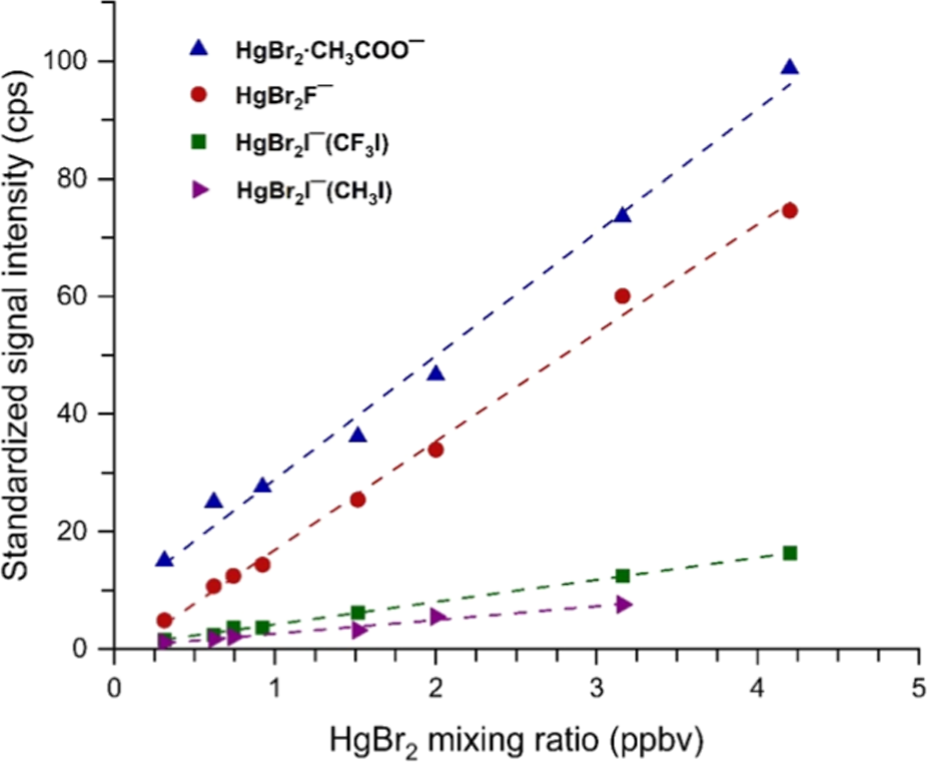
Experimental calibration curves obtained using selected ion monitoring, showing the dependence of standardized product ion intensity (corresponding to 1 Mcps reagent ion signal) on HgBr_2_ mixing ratio. Reagent ions SF_6_^–^, CH_3_COO^–^·CH_3_COOH, and I^–^ were used to form HgBr_2_F^–^, HgBr_2_CH_3_COO^–^, and HgBr_2_I^–^, respectively. The iodide reagent ion was produced from two different precursors, CF_3_I and CH_3_I, as indicated in the legend.

One can expect that the rate of the ion exchange reactions that produce thermalized ion products can be well described by the ion–molecule collision rates, such as those predicted by the ADO (or Langevin) theory. Then, for analyte mixing ratios below 100 ppbv, where reagent-ion depletion is negligible and pseudo-first-order ion–molecule reaction conditions apply, theoretical detection sensitivity (*S*_t_) can be calculated based on the ion–molecule reaction rate equation,^40,67^

4 $$S_{t}=R\times k\times t\times f_{iso}\times C_{1\;ppbv}\;\left(cps{\;ppbv}^{-1}\right)$$

where *R* is the reagent ion count rate, *k* is the ion–molecule reaction rate constant, *t* is the ion drift time, *f*_iso_ = 0.22 is the ratio of the relative intensity of the monitored isotope peak to the sum of relative intensities of all isotope peaks in the product ion, and *C*_1 ppbv_ = 8.42 × 10^7^ molecules cm^–3^ is the concentration of the analyte gas at a mixing ratio of 1 ppbv at a total pressure of 2.56 Torr and temperature of 298 K. Table 6 shows values of the ion–molecule reaction rate constants between reagent ions and HgBr_2_, as well as the parameters needed for the Langevin theory. Table 7 summarizes the theoretical sensitivities for HgBr_2_ detection using different reagent ions, together with the parameters used in these calculations. Table 5 shows agreement between the normalized theoretical and experimental sensitivities for CH_3_COO^–^·CH_3_COOH and SF_6_^–^, with the ratio of theoretical to experimental sensitivity being 1.3 in both cases. Some of this remaining discrepancy may be due to combined uncertainties in the calculated ion–molecule reaction rates and reduced ion mobilities, but the majority is caused by ion mass discrimination. Our instrument employs a 19 mm segmented quadrupole equipped with both prefilter and postfilter rods, where transmission efficiency is 41.2% at 131 amu and 29.2% at 414 amu.^68^ A factor of 1.4 lower transmission efficiency for the higher-*m*/*z* product ions nearly matches the 1.3-fold lower experimental sensitivity relative to theoretical sensitivity.

**Table 6:** Ion–molecule Rate Constants for the Reaction of HgBr_2_ with Several Reagent Ions Calculated by the Langevin Theory, along with Parameters Used for the Calculation

		α^b^	*k*_Lan_
reagent ion	μ_m_ ^a^	Å^3^	10^–10^ cm^3^ s^–1^ molecule^–1^
CH_3_COO^–^·CH_3_COOH	89.46	13.69	9.16
I^–^	93.85	13.69	8.94
SF_6_^–^	103.91	13.69	8.50

a Reduced mass of analyte and reagent ion.

b Isentropic component of the polarizability tensor.

**Table 7:** Theoretical Sensitivities for Detection of HgBr_2_ by Different Reagent Ions, along with the Reduced Ion Mobility, Electric Field Strength, and Reaction Time Used as Inputs to Eq 4

	μ^a^	*E* ^b^	*t*	*S*_t_ ^c^
reagent ion	cm^2^ V^–1^ s^–1^	V cm^–1^	ms	ncps ppb^–1^
I^–^	686.6	–21	1.52	25
CH_3_COO^–^·CH_3_COOH	555.8	–21	1.63	28
SF_6_^–^	706.4	–21	1.5	24

a Ion mobility at 298 K and 2.56 Torr, calculated using IMoS.

b Electric field in the drift tube.

c Normalized to 1 Mcps reagent ion signal intensity.

The sensitivity toward HgBr_2_ with SF_6_^–^ determined in this study using a permeation source is about an order of magnitude higher than the sensitivity toward HgCl_2_ with the same reagent ion in our previous study^30^ that employed a glass tube filled with solid HgCl_2_, indicating that the assumption of the fully saturated vapor was incorrect. The ratios of normalized theoretical to experimental sensitivity for the iodide ion produced by CF_3_I/N_2_ and CH_3_I/N_2_ are 6.6 and 10.7, respectively. The significantly lower experimental sensitivities are mainly due to insufficient thermalization of the product ion, HgBr_2_·I^–^, as discussed above. A better performance in the case of iodide generated from CF_3_I is due to a significant contribution from the ion exchange reaction with CF_3_I_2_^–^, where the product does not require thermalization.

The limit of detection (LOD) was calculated based on the standard deviation of the background signal measured with the calibration source bypassed to eliminate the residual HgBr_2_. With a 60 s integration time and assuming a signal-to-noise ratio of 3, the LOD was 216 pptv for CH_3_COO^–^·CH_3_COOH, 53 pptv for I^–^ produced from CF_3_I, 268 pptv for I^–^ produced from CH_3_I, and 102 pptv for SF_6_^–^ (Table 5). Although the limit of detection is directly related to sensitivity, it is also significantly influenced by the background signal. Therefore, the cleanliness of the system plays an important role in achieving a lower LOD. Another factor is the mass of the detected ion, because as the ion mass increases, the background noise contributed by unrelated ions typically decreases. This occurs because, at higher masses, the probability of naturally existing interfering molecules becomes progressively lower. Indeed, as Table 5 demonstrates, lower LODs are achieved using the iodide reagent ion produced from CF_3_I as a precursor, even when the sensitivity was lower than that with SF_6_. This improvement results from the cleaner CF_3_I-based background, which minimizes chemical noise and spectral interferences. In contrast, SF_6_^–^ exhibits a higher sensitivity but yields a worse LOD due to a broader chemical background, producing greater interference.

## 4 Conclusions

In this study, the use of the CH_3_COO^–^·CH_3_COOH and I^–^ reagent ions for the detection of gaseous mercuric halides was investigated theoretically and experimentally. The results show that the corresponding ion–molecule reactions occur as ion exchange and ion–neutral clustering. The acetate generally forms product ions in a higher yield than the iodide because its complexes with mercuric halides are 7 kcal/mol more stable and formed primarily through ion exchange, requiring no thermalization. On the contrary, most of the iodide–mercuric halide complexes are produced via clustering, which requires collisional thermalization to dissipate the energy released during complex formation. Such thermalization is inefficient under the low-pressure and low-humidity experimental conditions of our study. Accordingly, the normalized experimental sensitivities decrease in the order CH_3_COO^–^·CH_3_COOH, I^–^ (generated from CF_3_I), and I^–^ (generated from CH_3_I). The higher sensitivity obtained with the iodide generated from CF_3_I versus CH_3_I is attributed to the relatively large amount of CF_3_I_2_^–^ clusters present in that system that can react with HgX_2_ through ion exchange, whereas bare iodide generated from CH_3_I reacts solely through clustering. The discrepancy between theoretical and experimental sensitivities increases in the order CH_3_COO^–^·CH_3_COOH, I^–^ (generated from CF_3_I), and I^–^ (generated from CH_3_I) because as the reaction mechanism shifts from primarily ion exchange (CH_3_COO^–^·CH_3_COOH and CF_3_I_2_^–^) to primarily clustering (I^–^), the product ions require more thermalization. The Langevin theory, used here to calculate theoretical sensitivities, assumes that the product ions are fully thermalized, and it performs well for the cases involving ion exchange. However, in the case of the bare iodide, this assumption is not valid, explaining the observed significant discrepancy between experimental and theoretical results. Although iodide is less suitable than CH_3_COO^–^·CH_3_COOH or SF_6_^–^ for the detection of mercuric halides in the experiments with lower IMR pressures, at higher pressures the thermalization will occur more efficiently, becoming competitive with thermal decay at ambient pressure. Furthermore, at typical ambient humidities, when iodide forms clusters with water, the reaction with mercuric halides will switch from clustering to ion exchange, requiring no thermalization.^34^ If the product ion is stable and thermalized, our results show that theoretical and experimental sensitivities agree with each other. In such cases, theoretical sensitivity can be used with confidence to estimate the concentration of Hg-containing free radicals and transient molecules measured by CIMS, where calibration using standards is impossible or impractical.

The soft ionization capability of the iodide CIMS provides a powerful analytical tool that has been used for detecting atmospheric trace gases, such as hydroperoxides and bicyclic peroxy radicals.^69–71^ The current LOD of a few hundred pptv is sufficient for laboratory experiments focused on mercury chemistry, making iodide and acetate CIMS applicable for identifying the products of HgBr reactions with NO_2_ ^72^ and O_3_,^73^ whose kinetics have been studied by the Pulsed Laser Photolysis-Laser-Induced Fluorescence. This LOD may be sufficient for sampling from high-concentration sources, such as flue gas from the stacks of cement plants, coal-fired power plants, and garbage incinerators, where GOM concentration commonly reaches tens of μg m^–3^ (a few ppbv).^74,75^ A higher-pressure implementation of CIMS can significantly improve LOD through the combination of more efficient thermalization, lower analyte and reagent ion dilution in the IMR region, and longer IMR time. Additional improvements can be achieved by employing a high-efficiency atmospheric-pressure ion interface, as described by Junninen et al.,^76^ which guides ions more effectively, reducing ion losses associated with multistage pumping, as well as by using a flow-opposed drift tube to concentrate and extract product ions prior to sampling.^77,78^ Molecular oxygen was not explicitly added to the bath gas in this study because O_2_ is not expected to react with or significantly deplete the reagent ions based on previous studies.^34,79,80^ Furthermore, O_2_ is not anticipated to interfere with the formation of mercuric compound–reagent ion clusters and introduce detectable interferences.^30^ Using CIMS at ambient pressure may allow for the direct detection of the atmospheric GOM, skipping the preconcentration step, similar to the detection of the atmospheric hydroxyl radical, sulfuric acid, and highly oxidized organic molecules.^29,81–83^ Further research will be needed to elucidate and address possible matrix effects not present in laboratory studies, such as the reduction in sensitivity or changes in ion chemistry caused by abundant ambient species and worsening of the limit of detection from the increase in the background signal in the mass region of mercuric product ions.

## Supplementary Material



## Notes

Schematics of the ion drift–chemical ionization mass spectrometer, calibration setup, and mercuric halide semiquantitative generation sources; representative reagent-ion mass spectra; tuned-to-detuned signal ratio as a function of ion mass; QRRK dissociation rate calculations; ion mobility input parameters (Cartesian coordinates, van der Waals radii, Mulliken charges, Lennard–Jones, and electrostatic parameters); and optimized geometries, electronic energies, zero-point energies, and vibrational frequencies for all calculated species (PDF)
