# Supplementary material for: Detection of Gaseous Mercuric Halides Using Acetate and Iodide Chemical Ionization Mass Spectrometry
Source: Anal Chem. 2026 Feb 25;98(13):9753–63. doi: 10.1021/acs.analchem.5c07120 (PMC13063219; doi:10.1021/acs.analchem.5c07120)
Supplement: Supplementary file 1 [file ac5c07120_si_001.pdf]

Detection of Gaseous Mercuric Halides using Acetate and Iodide Chemical Ionization Mass Spectrometry

Mohammad Borna Bahramsari[1], Alexei F Khalizov[1,2]

[1]Department of Chemistry and Environmental Science, New Jersey Institute of Technology, Newark, New Jersey 07102, United States
[2]Department of Chemical and Materials Engineering, New Jersey Institute of Technology, Newark, New Jersey 07102, United States

Content:
1 Text sections
4 Tables
12 Figures

Corresponding author: khalizov@njit.edu

S1: calculation of the dissociation rate complex of $HgBr_2$ with iodide

The decay rate of "hot" $HgBr_2I^-$ was calculated using quantum Rice–Ramsperger–Kassel (QRRK) theory, similar to the approach of Lee et al. for iodide–formic acid clusters [1],

$$k_{decay} = \nu \frac{j!\,(j-m+s-1)!}{(j-m)!\,(j+s-1)!}$$

where

- ***s*** is the number of harmonic oscillators, estimated as half the vibrational modes of a non-linear polyatomic $HgBr_2I^-$ cluster (S= (3N-6)/2)
- ***m*** is the total thermal (translational, rotational and vibrational) energy of the newly formed cluster of $HgBr_2I^-$, obtained from the DFT calculation
- ***j*** is the combined thermal energy of the isolated $HgBr_2$ molecule and I^- ion (excluding the vibrational zero-point energy of $HgBr_2$)
- **ν** is the geometric mean of the vibrational normal modes of $HgBr_2I^-$

The input parameters, calculated decay rate, and net collision rate with the bath gas are summarized in Table S1. Collision rates were adapted from Lee et al. [1] and scaled to the system pressure of 2.56 Torr. For a more detailed explanation of the QRRK methodology, see Lee et al[1].

Table S1: Key parameters, dissociation rate, and collision rate used in the QRRK decay rate calculation

**Parameter**	**Value**
s	3
m	156
j	174
ν [s^{-1}]	2.43×10^{12}
k_{decay} [s^{-1}]	3.00×10^{10}
k_{collison} [s^{-1}]	7.58×10^{7}

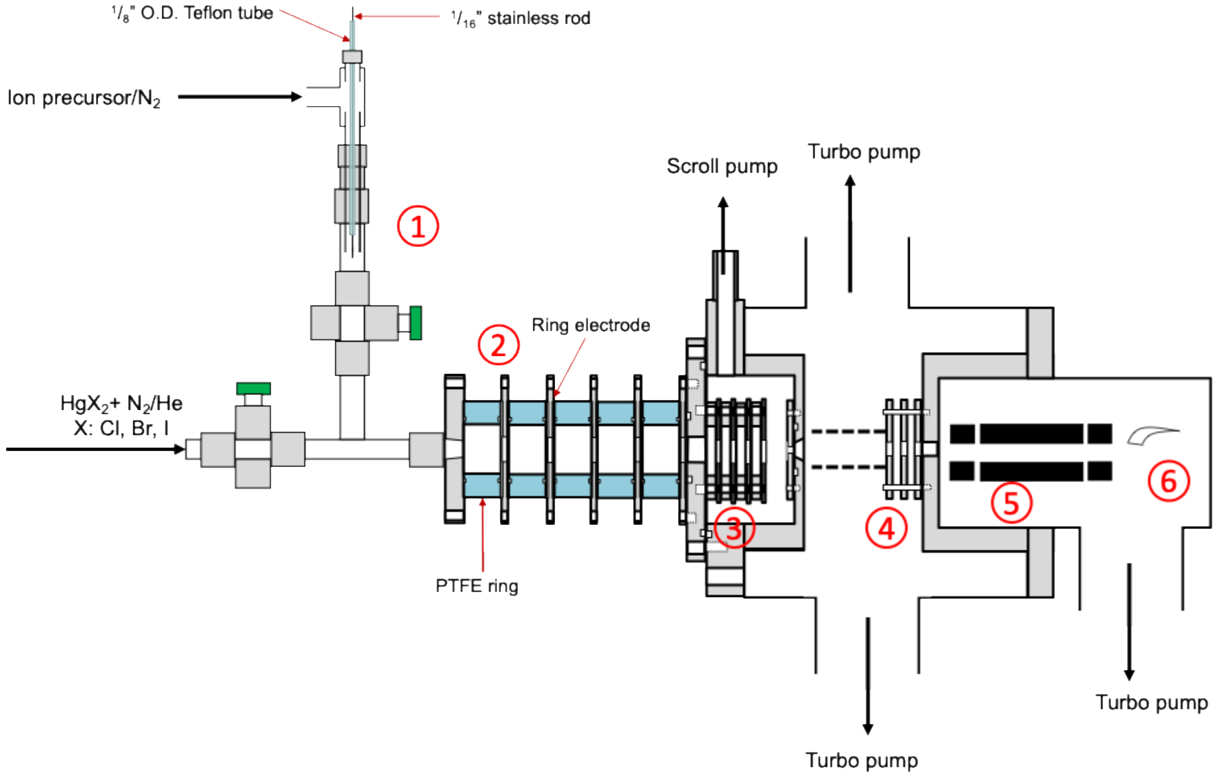

Figure S1: Schematic of the ion drift – chemical ionization mass spectrometer. The main components are: (1) corona discharge ion source, (2) drift tube, (3) collisional dissociation chamber (CDC), (4) focusing lenses, (5) quadrupole mass analyzer, and (6) electron multiplier detector.

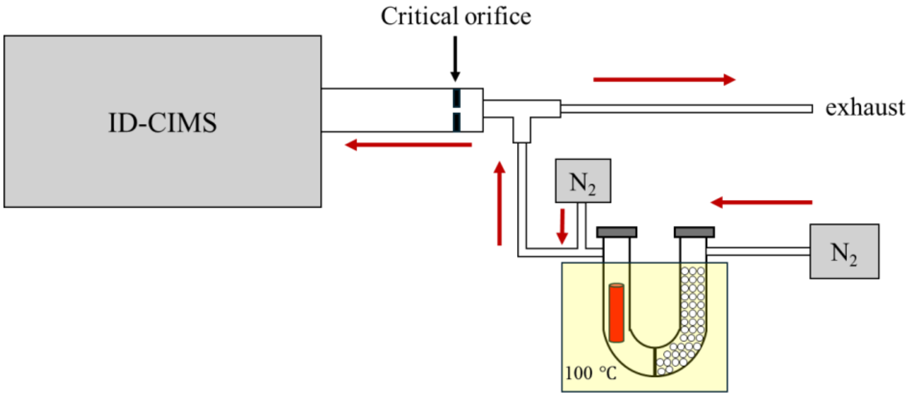

Figure S2: Calibration setup for quantitative detection of $HgBr_2$. The permeation source was held in a Pyrex glass U-tube maintained at 100 °C and atmospheric pressure. A critical orifice was used at the sampling entrance to the mass spectrometer to maintain a constant sample flow (0.3 slpm).

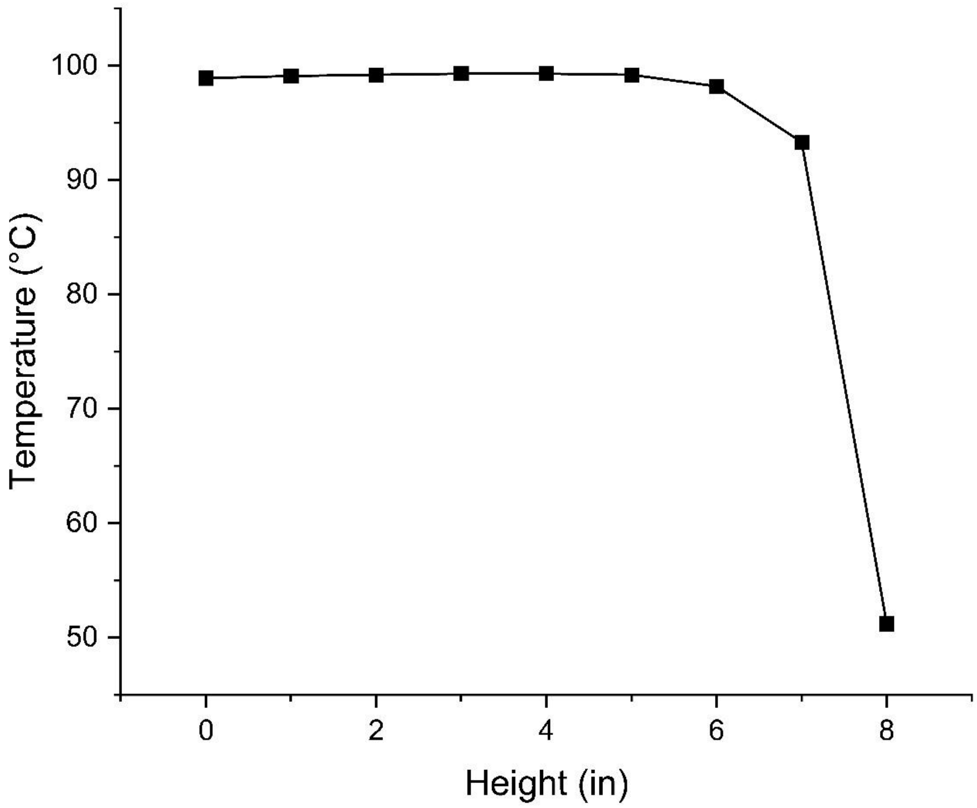

Figure S3: Vertical temperature profile measured from the base of the U-shaped glass tube arm that holds the permeation tube.

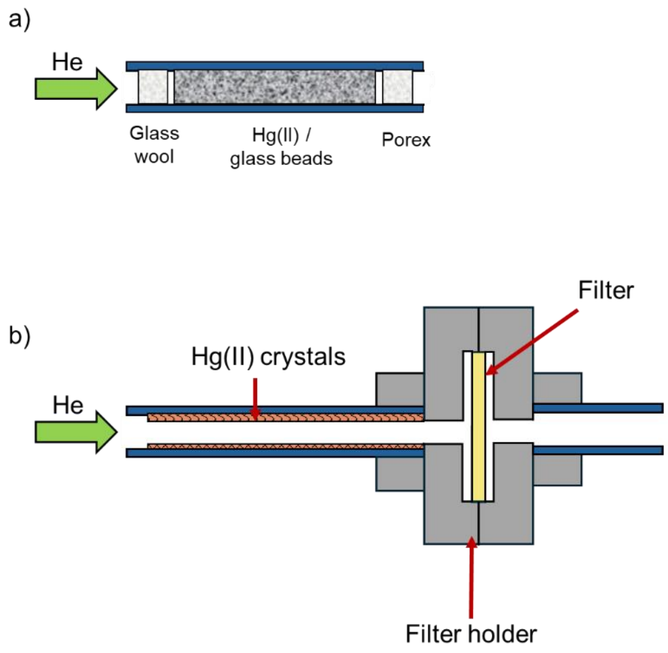

Figure S4: Schematics of the two sources for semi-quantitative generation of gaseous mercuric halides: (a) a packed bed made of glass beads coated with mercuric halide paste between plugs made of glass wool and Porex, (b) a glass tube coated on the inside with a mercuric halide solid layer and connected to a cartridge with a PTFE membrane filter

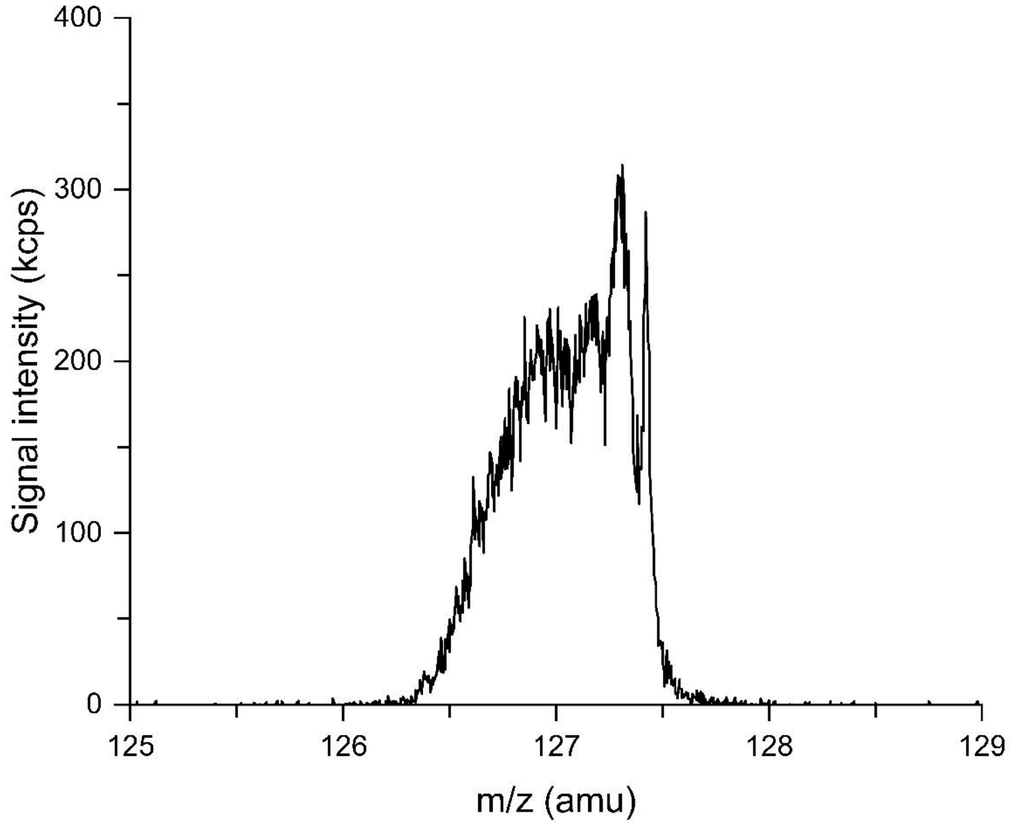

Figure S5: Mass spectrum of the iodide ion generated by corona discharge in CF_3I/N_2. Ion transmission was detuned to prevent the saturation of the multiplier. The estimated iodide signal intensity at the full multiplier voltage was 7.4 Mcps.

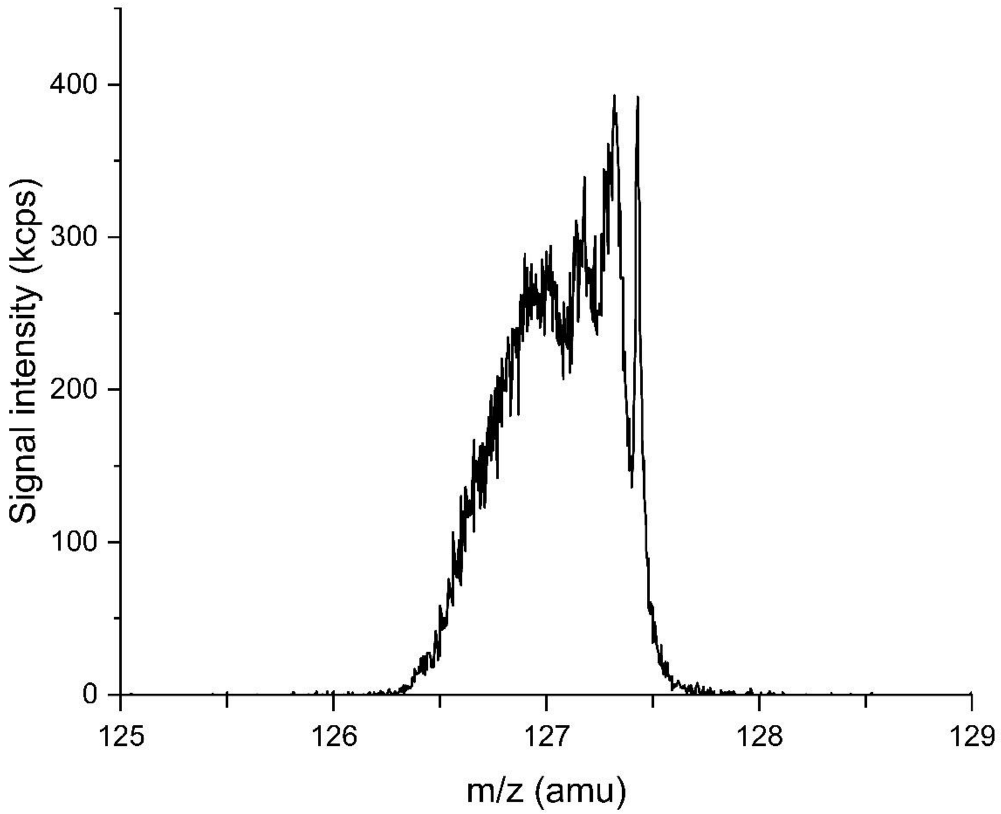

Figure S6: Mass spectrum of iodide generated by corona discharge in CH_3I/N_2. Ion transmission was detuned to prevent the saturation of the multiplier. The estimated iodide signal intensity at the full multiplier voltage is 8.9 Mcps.

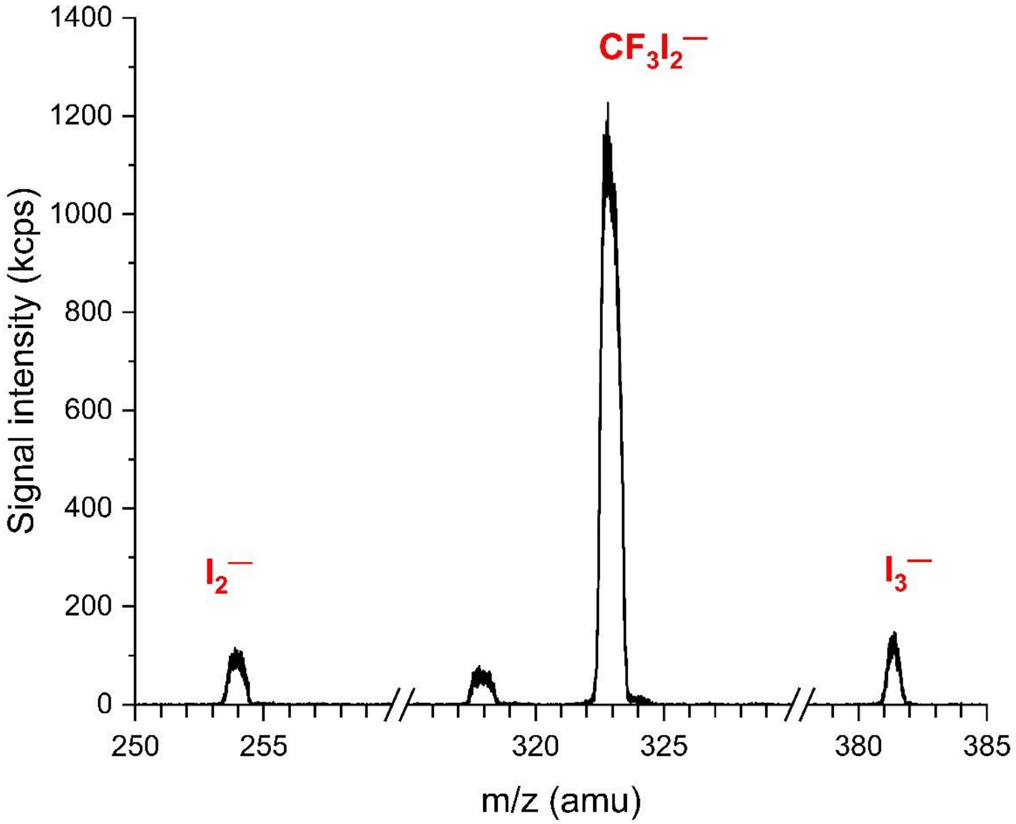

Figure S7: Mass spectrum of the major ions other than iodide generated by corona discharge in CF_3I/N_2 at the full multiplier voltage.

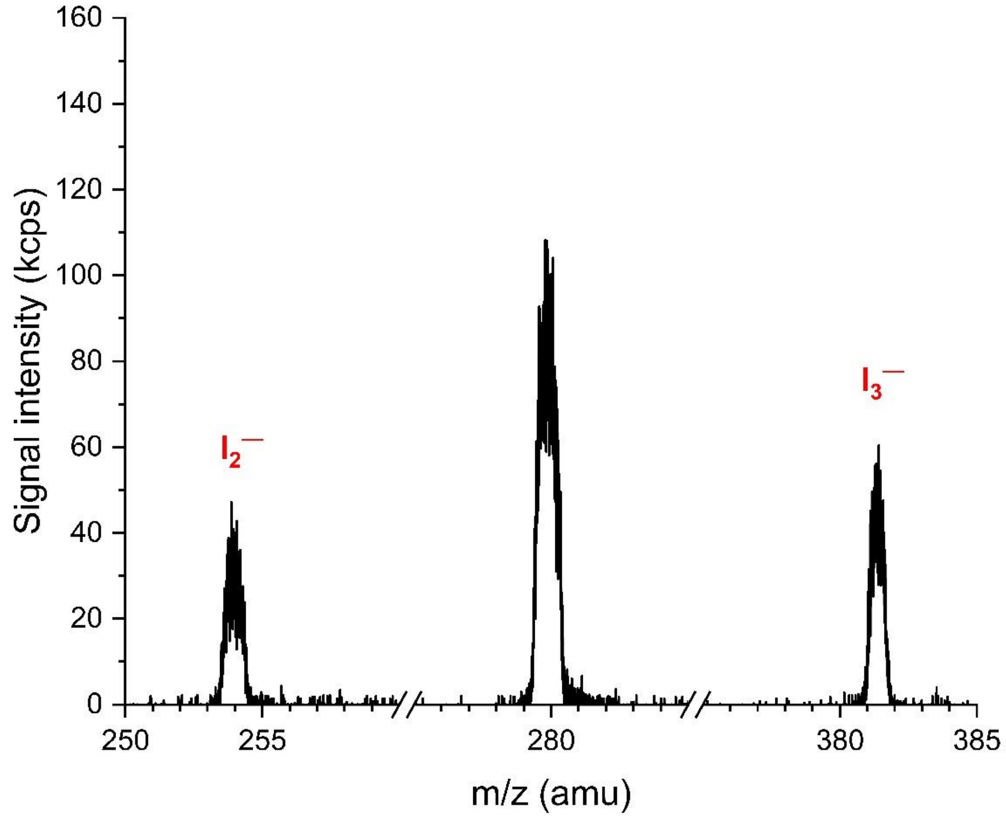

Figure S8: Mass spectrum of the major ions other than iodide generated by corona discharge in CH_3I/N_2 at the full multiplier voltage. We evaluated possible assignments for the signal at m/z = 280 amu, including reagent-ion clusters and background-related species; however, no chemically reasonable candidate was identified.

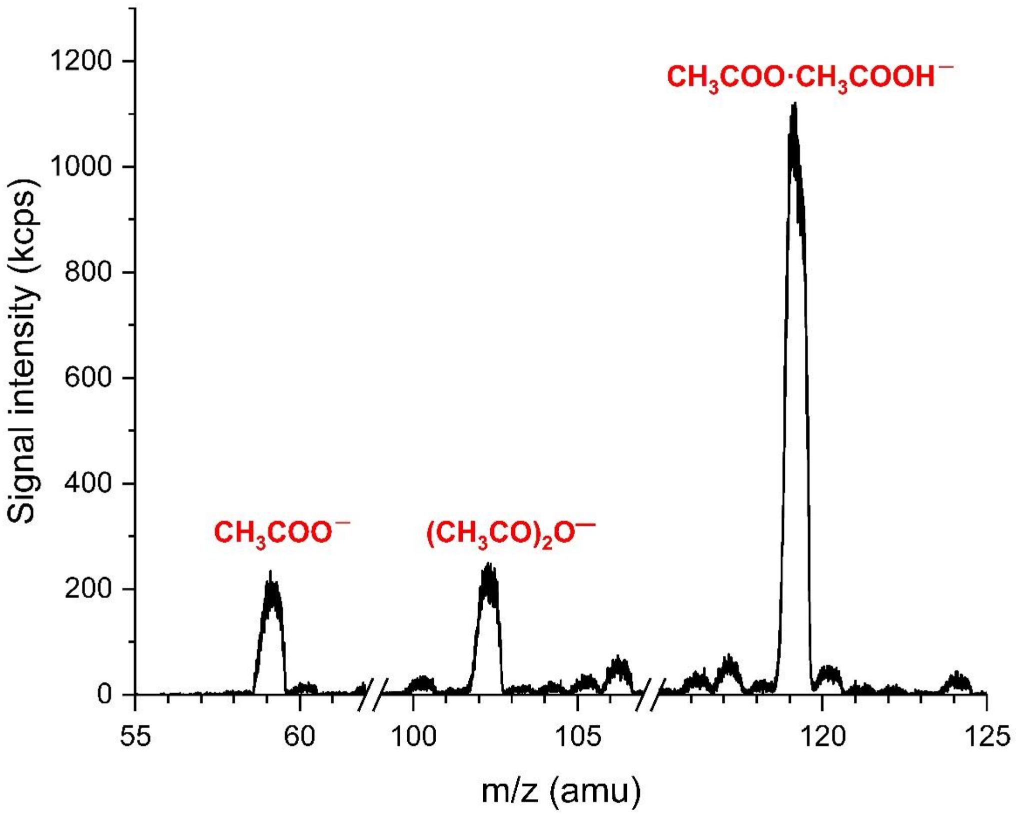

Figure S9: Mass spectrum of the ions generated by corona discharge in $(CH_3CO)_2O/N_2$ at the full multiplier voltage.

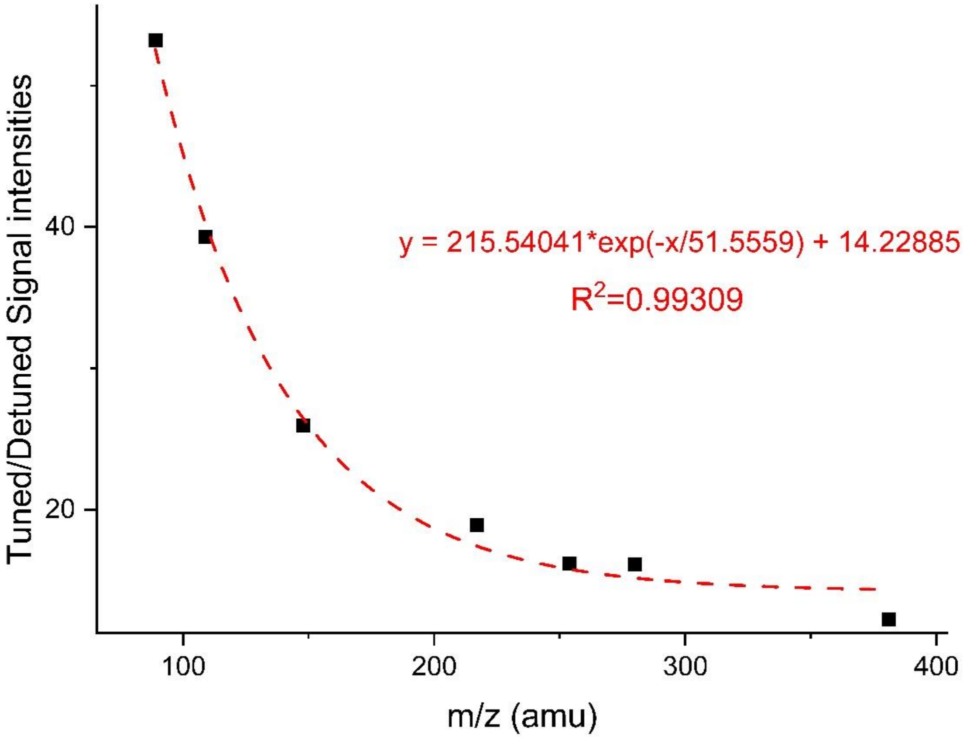

Figure S10: The ratio of tuned to detuned signal intensity as a function of ion mass (m/z). The dashed red curve represents an exponential fit to the experimental data.

Table S2: Optimized Cartesian coordinates (Å), van der Waals radii (Å), and Mulliken atomic charges for all ions for which ion mobility was calculated and used as input for the Ion Mobility Spectrometry Suite v1.13 (IMoS).

$SF6^{-}$

	X	Y	Z	Van der Waals radius (Å)	Mulliken charge
S	0	0	-0.00265	1.8	1.874638
F	0	0	1.619812	1.47	-0.404802
F	0	1.692938	0.063546	1.47	-0.471299
F	-1.69294	0	0.063546	1.47	-0.471299
F	1.692938	0	0.063546	1.47	-0.471299
F	0	-1.69294	0.063546	1.47	-0.471299
F	0	0	-1.86929	1.47	-0.584642

$NO_3^{-}\cdot HNO_3$

	X	Y	Z	Van der Waals radius (Å)	Mulliken charge
N	2.070761	-0.01925	0	1.55	0.868406
O	1.969133	-1.23802	-1.6E-05	1.52	-0.54041
O	3.137463	0.570614	0.000002	1.52	-0.64283
O	0.990804	0.694552	0.000014	1.52	-0.39527
H	-0.03833	-0.02576	0.000011	1.1	0.39223
N	-2.0745	0.02601	-1E-06	1.55	0.899354
O	-0.99051	-0.70062	0.000013	1.52	-0.40921
O	-3.13462	-0.56812	0.000003	1.52	-0.64021
O	-1.96421	1.238898	-1.6E-05	1.52	-0.53207

I^{-}

	X	Y	Z	Van der Waals radius (Å)	Mulliken charge
I	0	0	0	1.98	-1

H_3O^{+}

	X	Y	Z	Van der Waals radius (Å)	Mulliken charge
O	0	0	0.070539	1.52	-0.38153
H	0	0.947106	-0.188103	1.1	0.46051
H	0.820218	-0.473553	-0.188103	1.1	0.46051
H	-0.820218	-0.473553	-0.188103	1.1	0.46051

$CO3^{-}$

	X	Y	Z	Van der Waals radius	Mulliken charge
C	0.007873	0	-0.185195	1.7	0.508695
O	0.002971	0	1.047355	1.52	-0.591641
O	1.048282	0	-0.943371	1.52	-0.45853
O	-1.026477	0	-0.95162	1.52	-0.458524

$CH_3COO{\cdot}CH_3COOH^-$

	X	Y	Z	Van der Waals radius (Å)	Mulliken charge
C	-3.41253	-0.53837	0.05415	1.7	-0.62079
C	-2.07513	0.18782	-0.0363	1.7	0.368255
H	-3.33712	-1.49124	-0.4695	1.1	0.254704
H	-4.20231	0.066573	-0.38286	1.1	0.236433
H	-3.63808	-0.75829	1.097361	1.1	0.239863
O	-1.11329	-0.47559	0.511033	1.52	-0.50293
O	-1.99839	1.276378	-0.58403	1.52	-0.73785
H	-0.04572	-0.01759	0.448016	1.1	0.530516
H	3.641116	0.760438	1.100332	1.1	0.238703
O	1.110316	0.468151	0.510782	1.52	-0.49204
O	2.007131	-1.27458	-0.58755	1.52	-0.74972
C	2.071952	-0.18327	-0.03274	1.7	0.376944
C	3.414329	0.54214	0.05681	1.7	-0.6227
H	4.205103	-0.06107	-0.38177	1.1	0.229474
H	3.339166	1.496341	-0.46491	1.1	0.251138

Table S3: Lennard–Jones (ε and σ) parameters used in the ion mobility simulations. Values correspond to the default Trajectory Method parameter set implemented in IMoS for ion–neutral interactions with N_2 bath gas.

	ε ($J \times 10^{21}$)	σ (Å)
H	0.2518291316	1.8986165794
C	0.5725617712	3.2254869663
O	0.4327052508	3.0749947111
N	0.5270966235	3.5719061739
F	0.3950040443	3.0146504054
Cs	0.5814000000	4.2008100000
Na	0.4167763200	3.5000000000
Cl	0.4167763200	3.5000000000
I	0.6300000000	5.4000000000
K	0.4167763200	3.5000000000
Rb	0.4167763200	3.5000000000
P	0.4167763200	3.5000000000
Ca	0.4167763200	3.5000000000
Li	0.4167763200	3.5000000000
S	0.4167763200	3.5000000000
Other	0.4167763200	3.5000000000

Table S4: Optimized Cartesian coordinates (Å), total electronic energies, zero-point energies (ZPE), and harmonic vibrational frequencies (cm^{-1}) for all species considered in this study.

$HgBr_2$

	X (Å)	Y (Å)	Z (Å)	Energy	ZPE	frequencies (cm^{-1})
Hg	0	0	0	-987.5150021	0.001412	66.19, 66.19, 211.95, 275.43
Br	0	0	-2.41089			
Br	0	0	2.410887			

$HgCl_2$

	X	Y	Z	Energy	ZPE	frequencies (cm^{-1})
Hg	-1.25121	1.254193	0	-1073.84728	0.0019	97.55, 344.32, 392.33
Cl	1.024811	1.311083	0			
Cl	-3.52724	1.198044	0			

HgI_2

	X	Y	Z	Energy	ZPE	frequencies (cm^{-1})
Hg	-0.53347	1.562059	0	-744.7113795	0.000963	49.35, 152.29, 221.05
I	2.055166	1.541511	0			
I	-3.12209	1.58348	0			

$HgBr_2^-$

	X	Y	Z	Energy	ZPE	frequencies (cm^{-1})
Hg	0	0	0.52663	-987.5852023	0.000668	25.78, 131.58, 135.95
Br	0	2.50218	-0.60187			
Br	0	-2.50218	-0.60187			

$HgCl_2^-$

	X	Y	Z	Energy	ZPE	frequencies (cm^{-1})
Hg	0	0	0.346824	-1073.91405	0.000973	39.78, 186.64, 200.60
Cl	0	-2.32153	-0.81605			
Cl	0	2.321531	-0.81605			

HgI_2^-

	X	Y	Z	Energy	ZPE	frequencies (cm^{-1})
Hg	0	0	0.612903	-744.7852437	0.000485	16.26, 92.94, 103.86
I	0	2.74952	-0.46257			
I	0	-2.74952	-0.46257			

$(CH_3CO)_2O$

	X	Y	Z	Energy	ZPE	frequencies (cm^{-1})
C	2.337483	0.734693	0.337356	-381.7279031	0.099292	57.99, 69.32, 130.02, 131.59, 189.50,
C	1.188381	-0.08086	-0.16559			

H	3.269401	0.240647	0.085949			339.72, 427.93, 541.85, 566.02, 601.18, 688.47, 840.93, 933.48, 1025.89, 1033.03, 1070.70, 1082.47, 1183.92, 1259.58, 1401.32, 1407.52, 1472.60, 1472.99, 1479.82, 1480.69, 1871.23, 1943.69, 3085.92, 3086.07, 3154.03, 3154.04, 3200.29, 3200.40
H	2.246979	0.842615	1.4178			
H	2.299982	1.731694	-0.09772			
O	-0.000001	0.610387	-5E-06			
C	-1.18838	-0.08087	0.165587			
O	-1.2426	-1.14744	0.683305			
C	-2.33749	0.734684	-0.33735			
H	-2.29999	1.731686	0.097722			
H	-3.2694	0.240635	-0.08594			
H	-2.24699	0.842603	-1.4178			
O	1.242609	-1.14743	-0.68331			

$(CH_3CO)_2O^{-}$

	X	Y	Z	Energy	ZPE	frequencies (cm^{-1})
C	2.320431	-0.76168	-0.30925	-381.715348	0.100230	58.34, 81.91, 110.95, 124.74, 182.99, 339.12, 419.94, 532.59, 540.00, 635.75, 685.01, 828.96, 933.32, 1034.26, 1043.24, 1044.43, 1059.85, 1254.96, 1300.72, 1397.12, 1452.24, 1453.34, 1465.45, 1466.73, 1779.78, 1907.73, 2050.55, 3054.35, 3058.60, 3107.18, 3185.92, 3186.62, 3219.02
C	1.201659	0.138162	0.113489			
H	3.269804	-0.25543	-0.16844			
H	2.186999	-1.0439	-1.35293			
H	2.287117	-1.67955	0.279684			
O	-0.000002	-0.5302	-0.000001			
C	-1.20166	0.138163	-0.11349			
O	-1.29723	1.240957	-0.54968			
C	-2.32044	-0.76168	0.309252			
H	-2.28712	-1.67954	-0.27969			
H	-3.26981	-0.25543	0.16844			
H	-2.187	-1.0439	1.352924			
O	1.297228	1.240955	0.549679			

CH_3COO

	X	Y	Z	Energy	ZPE	frequencies (cm^{-1})
C	-1.34425	-0.11592	0	-228.3991077	0.047906	−17.14, 325.47, 463.67, 583.77, 862.96, 943.34, 1050.39, 1254.22, 1380.08, 1472.87, 1477.58, 1785.56, 3083.53, 3170.76, 3174.17
C	0.160336	-0.06301	0			
H	-1.79005	0.875873	0			
H	-1.65792	-0.66499	0.885374			
H	-1.65792	-0.66499	-0.88537			
O	0.916738	-0.99335	0			
O	0.609486	1.184345	0			

CH_3COO^{-}

	X	Y	Z	Energy	ZPE	frequencies (cm^{-1})
C	1.343622	0.049492	-0.00157	-228.525324	0.048585	32.62, 442.66, 621.01, 636.68, 892.98,
C	-0.2088	-0.00119	-0.00305			

H	1.709742	-0.38282	0.931957			1001.24, 1043.20, 1321.91, 1400.11, 1468.38, 1478.96, 1697.58, 3045.42, 3111.05, 3132.54
H	1.70987	1.070712	-0.09772			
H	1.729332	-0.56626	-0.81567			
O	-0.69712	-1.1515	0.000633			
O	-0.79761	1.100064	0.000513			

I_2

	X	Y	Z	Energy	ZPE	frequencies (cm^{-1})
I	0	0	1.329707	-591.2657538	0.000532	233.61
I	0	0	-1.32971			

I_2^{-}

	X	Y	Z	Energy	ZPE	frequencies (cm^{-1})
I	0	0	1.61247	-591.3643355	0.000245	107.55
I	0	0	-1.61247			

I_3

	X	Y	Z	Energy	ZPE	frequencies (cm^{-1})
I	2.98253	0.010726	0	-886.8765135	0.000615	-13.80 , 43.97, 225.837
I	0.309382	0.001482	0			
I	-3.29191	-0.01221	0			

I_3^{-}

	X	Y	Z	Energy	ZPE	frequencies (cm^{-1})
I	2.923679	0.00054	0	-887.0340117	0.00073	59.97, 119.74, 140.78
I	0	0.00028	0			
I	-2.92368	-0.00082	0			

$CH_3COO \cdot CH_3COOH^{-}$

	X	Y	Z	Energy	ZPE	frequencies (cm^{-1})
C	3.412526	0.53837	0.054149	-457.6561964	0.108627	21.82, 43.05, 51.73, 80.57, 84.68, 121.16, 127.61, 230.51, 375.50, 498.36, 606.48, 617.97, 620.13, 651.12, 769.64, 921.31, 937.81, 1026.36, 1039.02, 1061.38, 1063.62, 1350.62, 1352.38, 1393.65, 1433.87, 1457.65, 1473.96, 1474.96, 1481.46, 1481.90, 1520.22,
C	2.075132	-0.18782	-0.03631			
H	3.33712	1.491238	-0.4695			
H	4.202304	-0.06657	-0.38286			
H	3.638078	0.758289	1.097359			
O	1.113293	0.475594	0.511031			
O	1.998387	-1.27638	-0.58404			
H	0.04572	0.017588	0.448014			
H	-3.64112	-0.76044	1.100331			
O	-1.11032	-0.46815	0.51078			
O	-2.00713	1.274581	-0.58755			
C	-2.07195	0.183268	-0.03274			
C	-3.41433	-0.54214	0.056809			

H	-4.2051	0.061078	-0.38178			1757.86, 1817.45, 3066.90, 3070.22, 3133.87, 3137.26, 3161.25, 3166.54
H	-3.33917	-1.49634	-0.46492			

$HgBr_2 \cdot CH_3COO^-$

	X	Y	Z	Energy	ZPE	frequencies (cm^{-1})
C	0.000074	0.000034	-0.000001	-1216.111616	0.051637	19.00, 29.62, 38.12, 51.74, 69.03, 111.58, 122.91, 169.96, 197.63, 204.55, 454.39, 625.25, 685.63, 958.64, 1028.45, 1067.72, 1362.31, 1470.74, 1472.26, 1494.78, 1636.33, 3075.09, 3145.59, 3174.53.
C	0.000032	-0.000002	1.51869			
H	1.018272	-0.00022	-0.37997			
H	-0.55828	0.852018	-0.37905			
H	-0.49426	-0.91221	-0.33601			
O	0.974436	-0.52593	2.110309			
O	-0.98766	0.501969	2.109297			
Hg	-0.03907	-0.07021	4.291641			
Br	-1.15754	-2.20491	5.126213			
Br	1.045583	2.001361	5.30574			

$HgCl_2 \cdot CH_3COO^-$

	X	Y	Z	Energy	ZPE	frequencies (cm^{-1})
C	-0.00419	-0.00994	0.104169	-1302.443267	0.052131	27.78, 35.01, 54.09, 66.75, 69.04, 110.22, 120.70, 197.45, 270.91, 285.66, 453.55, 625.42, 684.32, 956.85, 1027.58, 1067.50, 1360.96, 1469.24, 1473.55, 1493.50, 1638.35, 3074.88, 3145.21, 3174.33.
C	-0.02191	-0.02558	1.623354			
H	0.961788	0.381345	-0.21721			
H	-0.78733	0.637544	-0.28087			
H	-0.10702	-1.02024	-0.28404			
O	0.485647	-1.01545	2.205787			
O	-0.51762	0.96067	2.220565			
Hg	0.019204	-0.03128	4.403165			
Cl	-1.99232	-1.05958	5.255886			
Cl	2.056703	0.999015	5.18981			

$HgI_2 \cdot CH_3COO^-$

	X	Y	Z	Energy	ZPE	frequencies (cm^{-1})
C	0.000785	-0.00689	0.091375	-973.3091962	0.05139	18.38, 26.27, 30.82, 42.22, 63.98, 107.16, 122.05, 127.37, 155.50, 204.85, 453.57, 626.82, 686.01, 959.49, 1027.92, 1069.27, 1363.33, 1470.49, 1473.04, 1495.35, 1632.90, 3076.90, 3147.65, 3176.23.
C	-0.01794	-0.02758	1.609054			
H	0.945331	0.435823	-0.22678			
H	-0.81164	0.606163	-0.28965			
H	-0.0546	-1.01826	-0.30246			
O	0.454658	-1.03324	2.193931			
O	-0.48045	0.973426	2.209757			
Hg	0.017343	-0.03298	4.378113			
Cl	-2.24516	-1.09579	5.454069			
Cl	2.319729	1.03213	5.367699			

$HgBr_2 \cdot I^-$

	X	Y	Z	Energy	ZPE	frequencies (cm^{-1})
Hg	0.013856	0.05223	0.526237	-1283.291535	0.001384	33.26, 38.70, 54.82, 133.83, 171.71, 175.24
Br	-0.00854	2.240012	-0.85422			
Br	-0.00646	-2.2128	-0.72348			
I	0.058445	0.132885	3.272562			

$HgCl_2 \cdot I^-$

	X	Y	Z	Energy	ZPE	frequencies (cm^{-1})
Hg	-2.26772	1.871892	0.008023	-1369.623253	0.001862	39.03, 53.62, 69.19, 137.37, 253.00, 265.11
Cl	-4.41346	0.670812	-0.00258			
Cl	-0.21106	0.523955	-0.00376			
I	-2.17215	4.606572	0.032025			

$HgI_2 \cdot I^-$

	X	Y	Z	Energy	ZPE	frequencies (cm^{-1})
Hg	0	-0.00028	0	-1040.48862	0.001169	32.22, 32.24, 47.97, 122.55, 138.98, 139.01
I	0	0.000075	2.758111			
I	2.388594	0.000075	-1.37906			
I	-2.38859	-0.00099	-1.37906			

$HgBr_2 \cdot I_2^-$

	X	Y	Z	Energy	ZPE	frequencies (cm^{-1})
Hg	0.022378	-0.15409	0.224344	-1578.917557	0.001725	25.85, 26.03, 36.54, 40.14, 55.10, 67.47, 115.94, 176.06, 214.26
Br	0.009749	2.300159	-0.34601			
Br	-0.04702	-2.46729	-0.77222			
I	1.782518	-0.40677	2.81633			
I	-1.45745	-0.38354	2.988012			

$HgCl_2 \cdot I_2^-$

	X	Y	Z	Energy	ZPE	frequencies (cm^{-1})
Hg	1.20251	0	0	-1665.249781	0.002330	31.63, 33.48, 46.95, 55.72, 70.39, 74.24, 115.69, 282.24, 312.24
Cl	1.86841	0	2.28445			
Cl	1.86841	0	-2.28445			
I	-1.50686	1.62202	0			
I	-1.50686	-1.62202	0			

$HgI_2 \cdot I_2^-$

	X	Y	Z	Energy	ZPE	frequencies (cm^{-1})

Hg	0.118497	0.13862	0.707834	-1336.114403	0.001463	20.83, 24.18, 31.73, 32.42, 48.65, 68.12, 116.29, 130.02, 169.73
I	0.06743	2.619111	-0.34989			
I	-0.01976	-2.45056	-0.03462			
I	2.011568	0.261475	3.206898			
I	-1.21375	0.337748	3.54149			

$HgBr_2 \cdot I_3^-$

	X	Y	Z	Energy	ZPE	frequencies (cm^{-1})
Hg	-2.22031	0.412467	0.000002	-1874.57649	0.002264	1.46, 10.69, 31.16, 34.35, 53.43, 53.45, 57.40, 88.23, 101.67, 168.25, 179.82, 213.69
Br	-0.9763	2.593307	-0.000015			
Br	-4.58276	-0.4889	0.000031			
I	-0.41512	-1.86695	-0.00002			
I	2.42915	-0.59227	-0.000003			
I	5.008458	0.446934	0.000015			

$HgCl_2 \cdot I_3^-$

	X	Y	Z	Energy	ZPE	frequencies (cm^{-1})
Hg	1.774995	0.900299	0.000003	-1960.908976	0.002980	6.76, 18.57, 46.63, 52.92, 59.31, 61.24, 75.84, 85.59, 109.48, 162.27, 291.47, 327.92
Cl	1.944026	1.408208	-2.29315			
Cl	1.943993	1.408222	2.293155			
I	-3.87164	0.598206	-0.000015			
I	-1.38671	-0.72019	-0.000003			
I	1.331988	-2.14036	0.00001			

$HgI_2 \cdot I_3^-$

	X	Y	Z	Energy	ZPE	frequencies (cm^{-1})
Hg	-0.7319	0.21317	0.423669	-1631.773389	0.002051	9.88, 11.40, 29.80, 32.81, 44.66, 57.08, 57.23, 74.78, 105.95, 132.44, 168.50, 175.66
I	-0.17668	2.68998	-0.44539			
I	-0.56278	-2.37992	-0.2437			
I	3.413061	0.060977	4.532501			
I	0.731094	0.235959	3.79701			

CF_3I

	X	Y	Z	Energy	ZPE	frequencies (cm^{-1})
C	-1.17545	0.00049	0.0002	-633.28576	0.014305	271.39, 271.87, 296.37, 544.74, 544.84, 762.99, 1105.01, 1240.47, 1241.40
F	-1.64017	-0.16271	1.22954			
I	0.9688	-0.00037	-0.00019			
F	-1.64099	-0.98221	-0.75575			
F	-1.64038	1.14679	-0.47278			

$CF_3I_2^-$

	X	Y	Z	Energy	ZPE	frequencies (cm^{-1})
C	-2.80718	-0.00076	0.0001	-929.0329901	0.014072	53.36, 54.45, 78.87, 245.82, 258.04, 258.24, 533.14, 533.18, 736.77, 1127.54, 1148.68, 1148.95
F	-3.326	-0.01303	1.24138			
F	-3.32532	-1.07036	-0.63051			
F	-3.32742	1.07951	-0.61018			
I	-0.62094	0.00123	-0.00021			
I	2.63323	-0.00049	0.00008			

References

[1] B.H. Lee, F.D. Lopez-Hilfiker, C. Mohr, T. Kurtén, D.R. Worsnop, J.A. Thornton, An Iodide-Adduct High-Resolution Time-of-Flight Chemical-Ionization Mass Spectrometer: Application to Atmospheric Inorganic and Organic Compounds, Environ. Sci. Technol., 48 (2014) 6309-6317.
